# Can Lunar and Martian Soils Support Food Plant Production? Effects of Horse/Swine Monogastric Manure Fertilisation on Regolith Simulants Enzymatic Activity, Nutrient Bioavailability, and Lettuce Growth

**DOI:** 10.3390/plants11233345

**Published:** 2022-12-02

**Authors:** Antonio G. Caporale, Mariana Amato, Luigi G. Duri, Rocco Bochicchio, Stefania De Pascale, Giuseppe Di Rauso Simeone, Mario Palladino, Antonio Pannico, Maria A. Rao, Youssef Rouphael, Paola Adamo

**Affiliations:** 1 Department of Agricultural Sciences, University of Naples Federico II, 80055 Portici, Italy; 2 School of Agriculture, Forestry, Food and Environmental Sciences, University of Basilicata, 85100 Potenza, Italy

**Keywords:** space farming, Mars and Lunar simulants, organic amendment, sustainable use of resources, extra-terrestrial food production, bioregenerative life support systems

## Abstract

To make feasible the crewed missions to the Moon or Mars, space research is focusing on the development of bioregenerative life support systems (BLSS) designed to produce food crops based on in situ resource utilisation (ISRU), allowing to reduce terrestrial input and to recycle organic wastes. In this regard, a major question concerns the suitability of native regoliths for plant growth and how their agronomic performance is affected by additions of organic matter from crew waste. We tested plant growth substrates consisting of MMS-1 (Mars) or LHS-1 (Lunar) simulants mixed with a commercial horse/swine monogastric manure (i.e., an analogue of crew excreta and crop residues) at varying rates (100:0, 90:10, 70:30, 50:50, *w*/*w*). Specifically, we measured: (i) lettuce (*Lactuca sativa* L. cultivar ‘Grand Rapids’) growth (at 30 days in open gas exchange climate chamber with no fertilisation), plant physiology, and nutrient uptake; as well as (ii) microbial biomass C and N, enzymatic activity, and nutrient bioavailability in the simulant/manure mixtures after plant growth. We discussed mechanisms of different plant yield, architecture, and physiology as a function of chemical, physico-hydraulic, and biological properties of different substrates. A better agronomic performance, in terms of plant growth and optically measured chlorophyll content, nutrient availability, and enzymatic activity, was provided by substrates containing MMS-1, in comparison to LHS-1-based ones, despite a lower volume of readily available water (likely due to the high-frequency low-volume irrigation strategy applied in our experiment and foreseen in space settings). Other physical and chemical properties, along with a different bioavailability of essential nutrients for plants and rhizosphere biota, alkalinity, and release of promptly bioavailable Na from substrates, were identified as the factors leading to the better ranking of MMS-1 in plant above and below-ground mass and physiology. Pure Mars (MMS-1) and Lunar (LHS-1) simulants were able to sustain plant growth even in absence of fertilisation, but the amendment with the monogastric manure significantly improved above- and below-ground plant biomass; moreover, the maximum lettuce leaf production, across combinations of simulants and amendment rates, was obtained in treatments resulting in a finer root system. Increasing rates of monogastric manure stimulated the growth of microbial biomass and enzymatic activities, such as dehydrogenase and alkaline phosphomonoesterase, which, in turn, fostered nutrient bioavailability. Consequently, nutrient uptake and translocation into lettuce leaves were enhanced with manure supply, with positive outcomes in the nutritional value of edible biomass for space crews. The best crop growth response was achieved with the 70:30 simulant/manure mixture due to good availability of nutrients and water compared to low amendment rates, and better-saturated hydraulic conductivity compared to high organic matter application. A 70:30 simulant/manure mixture is also a more sustainable option than a 50:50 mixture for a BLSS developed on ISRU strategy. Matching crop growth performance and (bio)chemical, mineralogical, and physico-hydraulic characteristics of possible plant growth media for space farming allows a better understanding of the processes and dynamics occurring in the experimental substrate/plant system, potentially suitable for an extra-terrestrial BLSS.

## 1. Introduction

Research on plants in space is shifting from cell biology to the production of crops, and from small-scale studies on synthetic growth media to investigations on materials relevant to Mars and Moon environments where crop growth is envisaged [1,2,3,4,5,6].

Fresh plants for crew consumption were first conceived within a very fast production timeframe as in the growing of sprouts for their nutritional and nutraceutical properties [7], but with the drawbacks of high use of resources. The idea of longer space missions and permanence on Mars and the Moon elicited the concept of bioregenerative life support systems (BLSS), where plants are grown with the aim of self-sufficiency for inputs, and where added functions are oxygen production and crew waste recycling for the recovery of water and nutrients [8]. An efficient and sustainable BLSS would be developed on the concept of in situ resource utilization (ISRU), which requires the use of native materials, such as regolith and waste as primary resources [9].

In their review on plant research in ground settings and during American, Asian, and European space missions, Zabel et al. [10] and Wheeler [8] listed more than 20 species grown in different space devices. These include food and ornamental crops, or plants solely devoted to research such as *Arabidopsis*. Edible species are cereals, legumes, and a range of tuber or vegetable crops [11,12] chosen for health and psychological benefits associated with growing plants in confined environments.

Due to palatability and the content of nutraceuticals, salad species enjoyed popularity to the point that plant-producing installations in space were referred to as “salad machines” since early efforts, as reviewed by Wheeler et al. [13]. Leafy vegetables also meet other major criteria for the choice of space crops, such as fast growth, high harvest index, and minimal area requirements.

Among recent research, Khodadad et al. [14] grew red romaine lettuce (*Lactuca sativa* cv. *Outredgeous*) in the “Veggie” growth system [15] on the International Space Station (ISS) in comparison with ground-grown plants. Space and ground leaves showed differences in some nutrients (Fe, K, Na, P, S, and Zn) and total phenolics, but no differences in anthocyanin and oxygen radical absorbance capacity (ORAC) levels. The growth medium of veggie chambers is a “pillow” made of solid porous argillite fertilised with nutrient solutions or controlled release fertiliser; therefore, all materials are sourced on Earth. Wheeler et al. [13] and Wheeler [8] reported that plants were grown in space on different artificial solid media and nutrient film techniques. Zabel et al. [10] reviewed growth media for on-orbit plant growth chambers, ranging from solidified agar to perforated tubing wrapped in a wick or porous tubes.

The quest for self-sufficiency and the BLSS concept raised interest for using growth media materials that may be locally sourced in space missions [16]. Research on such materials is also relevant to future in situ crop growing, for instance, on Mars and the Moon, and is effectively a first step for studying their potential agriculture environment. Gilrain et al. [2] proposed mixtures of compost and regolith analogue to Mars surface materials (referred to as simulants) for growing Swiss chard; Mortley et al. [3] and Wamelink et al. [4] tested Moon and Mars simulants as plant growth media. After a test on different plants [17], Fackrell [1] grew the legume moth bean (*Vigna aconitifolia*) on Mars simulant with a set of microbial inoculants to provide biologically fixed nitrogen and help acquisition of other nutrients.

As for lettuce, Caporale et al. [5] grew two varieties of lettuce on a coarse-textured alkaline Mars simulant (i.e., MMS-1) with the addition of organic matter from green compost, which may be produced in space from inedible parts of plants. The MMS-1 simulant was found to contain considerable amounts of Na and plant nutrients, such as Ca, Mg, and K, but no organic matter and related macronutrients needed for plant growth, such as N, available P, and S. Compost amendment complemented the simulant’s ability to provide elements for plant growth, lowered the bulk density and pH of the simulant, and modified its hydraulic properties. Organic amendment also contributes to enrich simulants in microbial biomass with its own activity and functionality useful to support nutrient bioavailability and organic matter breakdown [18]. This resulted in an improved growth of lettuce up to the dose of 30% green compost [5]. This research team also studied the lettuce nutritional profile, content of nutraceuticals, photosynthetic activity, and water use efficiency as a function of different MMS-1 simulant/green compost mixtures [6].

Optimisation of scarce resources, such as selected plant nutrients and water in space, required crop design to address allometric relations between above-ground plant parts. The increase in harvest index was targeted [13,19] by choosing appropriate species or creating dwarf varieties, such as the short life cycle of Apogee and Perigee wheats [20]. Allocation of resources to roots, though, received little attention in space plant research. Roots often represent an investment in inedible parts, but at the same time play a key role in acquiring resources and in driving below-ground processes [21]. In poor growth media, ratios of root to shoot mass are typically larger [22,23] and roots have a different architecture and fine root percentage [24,25], which increases the resource acquisition. Therefore, root/shoot ratios and root architecture represent a classical optimisation problem and need to be addressed for conditions relevant to crop production in space.

To enhance the fertility and physicochemical properties of the alkaline and nutrient-poor Lunar or Mars regolith simulants, potentially exploitable as plant growth media in BLSS, the amendment with stable organic matter (e.g., a monogastric-based manure, similar to composted plant residues and crew excreta, which can be produced during space missions), can be a viable and sustainable option, deserving scientific attention and experimentation.

In this context, the present study aims to evaluate the agronomic and environmental performances of Mars MMS-1 or Lunar LHS-1 simulants mixed, at increasing rates, with a commercial horse/swine monogastric manure (100:0, 90:10, 70:30, 50:50, *w*/*w*), to study as well substrate-related mechanisms underlying yield, architecture, and the physiology of lettuce plants (*Lactuca sativa* L. cultivar ‘Grand Rapids’) grown in these mixtures for 30 days, in an open gas exchange climate chamber with no fertilisation. This goal is relevant for viable production in BLSS, but also for giving insights on the possible exploitation of Mars and Moon regoliths for agricultural purposes.

We specifically determined lettuce growth, physiology, and nutrient uptake, as well as microbial biomass C and N, enzymatic activities, and nutrient bioavailability in simulant/manure mixtures after plant growth in order to test the following hypotheses:

(i) The mineralogical, physico-hydraulic and (bio)chemical properties of Lunar or Martian simulant-based substrates strongly affect their own capacity to support the growth and quality of lettuce plants;

(ii) The monogastric manure-based amendment relieves nutritional and physical constraints of pure mineral simulants for plant growth, allowing for production without fertilisation in a dose-dependent way. Manure enriches them in organic matter and microbial biomass that is able, in turn, to break down organic matter and make nutrients available. Microbial functionality should improve in terms of activity of the main soil enzymes involved in biogeochemical cycles of nutrients;

(iii) Plants growing on amended and pure regoliths modulate their investment in below-ground parts and their root morphology and topology according to nutrient availability of growth media, in order to optimise the global harvest index, understand plant behaviour and acquire information for crop design in regolith-based regenerative agriculture;

(iv) Organic matter supply by monogastric manure stimulates root growth and microbial activities in the rhizosphere, with positive outcomes on nutrient bioavailability and geochemistry.

## 2. Results and Discussion

### 2.1. Biometric Parameters of Lettuce Plant

The mean effect of simulant showed significantly higher values of GI, LN, LA, and dry biomass in MMS-1 compared to LHS-1, whereas DM was higher in the Lunar simulant (Table 1). The interaction between simulant (S) and amendment percentage (M) factors was statistically significant for GI, LN, LA, dry biomass, and DM (Table 1). Lettuce plants grown on the MMS-1 mixture with 30% manure recorded significantly higher values of GI, LN, LA, and dry biomass compared to pure MMS-1 simulant (52-, 2-, 28-, and 12-fold more than pure simulant, respectively) (Table 1). Similarly, within the different LHS-1-based mixtures, both 10% or 30% manure concentrations show significantly higher values of GI, LN, LA, and dry biomass than the pure LHS-1 simulant (on average 45-, 2-, 30-, and 8-fold more than pure simulant, respectively) (Table 1). In contrast, the leaf DM content was on average significantly higher by 94% and 112%, respectively, in pure MMS-1 and LHS-1 compared to the respective manure-treated mixtures (Table 1). In particular, this latter parameter, regardless of the simulant, turns out to be inversely correlated to the manure doses (R = 0.88).

Regolith simulants are extremely poor in both nutrients and organic matter, proving to be notably unsuitable for plant growth [26]; therefore, especially under such extreme conditions and in the absence of external nutrient inputs, organic amendment is particularly effective in improving simulant fertility [5,6,27]. In the present experiment, the higher growth of plants cultivated on the Mars simulant was probably due to the worse physico-chemical characteristics of the Lunar substrate (Table S1—Supplementary Material and [28]). Amendment treatments significantly promoted plant biometric characteristics compared to pure substrates, and in the range 0–30%, plant growth increased with manure dose. This result was ascribable to the improvement in hydraulic characteristics and nutrient availability driven by the manure supply [29], while the decrease in dry biomass recorded at the 50% dose could be due to a higher electrical conductivity and endowment of phytotoxic elements, such as Na [30]. Similar results were found in a previous work with MMS-1 and increasing doses of a green compost [6]. The decrease in leaf DM observed as the dose of manure increases may be ascribed to a higher water content in lettuce leaves probably related to greater water availability in the substrate resulting from the higher water holding capacity of the amended simulants (Table S1—Supplementary Material and [28]). In this regard, the DM content of plants grown under conditions of reduced water availability was found to increase likely as a result of higher accumulation of assimilates required for maintenance of plant metabolism and activation of stress responses [31].

Regarding root traits, the mean effect of simulants (Table 2) on Rdw, RA, and RL showed significantly higher values in MMS-1 with increases of about 100 to 200% compared to LHS-1. Differences were not significant for RV and SRS. Manure concentration as a main effect showed highest values at 30% and lowest at 0% for Rdw, RA, RL, and RV, with maximum differences of one order of magnitude. The specific root surface at 10% was about two-fold that of pure simulant and of other manure concentrations, which did not show significant differences among them.

**Figure 1:**
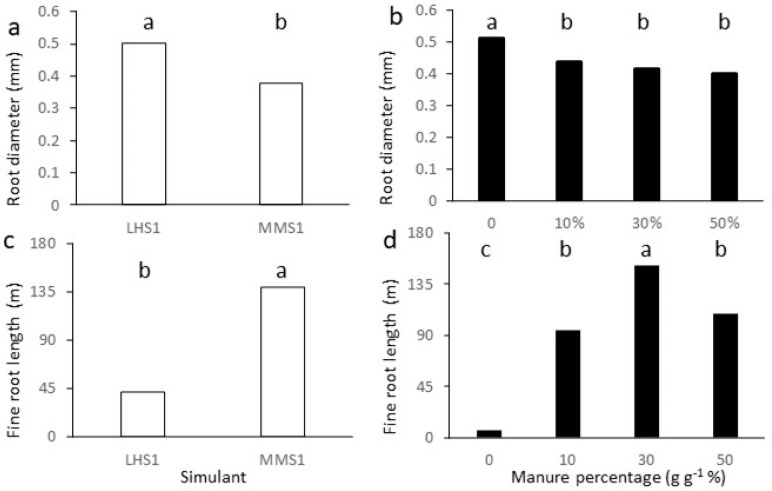
Main effects of simulant (**a**,**c**) and manure concentration (**b**,**d**) on root diameter (**a**,**b**) and length of roots with diameter < 0.5 mm (**c**,**d**). Bars with different letters are different for *p* < 0.05 at the post hoc Duncan’s mean separation test.

Interactions between experimental factors were significant for all root traits (Table 2), except diameter (Figure 1). For Rdw, RA, and RL, the highest value was found in MMS-1 at 30% manure, but for RA, this was not significantly different than in MMS-1 at 50%. Values were highest at 30% in both simulants for RV; for SRS values were highest in LHS-1 at 10%. Pure simulants always showed the lowest values, except for SRS in MMS-1, where values at 0% and 50% were not significantly different. For all traits reported in Table 2, values recorded in MMS-1 were in most cases statistically higher than those recorded in LHS-1 with equal manure percentage. Root dry mass in our work ranged from 0.05 g in LHS-1 at 0% manure to 1.46 g in MMS-1 at 30% manure. Dry mass values in pure simulants are lower than 0.1 g and lower than values reported in the literature for lettuce grown in different systems (substrate, hydroponics ore aeroponic—[32]) whereas amendment brings root dry mass closer to literature ranges [32,33]. Root traits in lettuce were reported to vary strongly with genetics and management. Our data are lower than values of about one to two thousand meters plant^−1^ reported by Murakami et al. [34] for field-grown lettuce, but higher than those found in Li et al. [32] in soilless systems and using an imaging system of lower resolution.

Root average diameters were higher in LHS-1 than in MMS-1 at all manure concentrations (Figure 1a), and at 0% manure in both simulants, whereas differences were not significant between 10%, 30%, and 50% manure (Figure 1b). Values of average diameter of 0.5 mm, such as in LHS-1, are in line with those reported by Li et al. [32] for different growth systems. Rowse [35] reported higher values in the uppermost 10 cm soil layer at harvest, while deeper roots were finer on average. Additionally, irrigation resulted in finer root diameter.

Absolute values of length for very fine roots (diameters smaller than 0.5 mm—Figure 1c,d) show large differences between treatments: average values of MMS-1 (Figure 1c) were 235% higher than those of LHS-1. Regarding the effect of manure, very fine root length increased from 15 to 24 times, with amendment reaching the highest value at 30% manure, and thereafter decreasing so that the length of very fine roots was not significantly different at 10 and 50% manure levels (Figure 1d). The percentage of root length allocated to each diameter class is reported in Figure S1 of Supplementary Material. Most of the root length was found in the finest root classes, with about 64 to 89% of roots in the class of diameter up to 0.5 mm (Figure S1a), about 9 to 29% in the class of roots with diameters between 0.5 and 1 mm (Figure S1b), and up to about 4% in the 1 > D < 1.5 mm class (Figure S1c). In the six classes with diameters from 1.5 to 4.5 mm, very small percentages were found, and trends of differences between treatments were similar between classes; we therefore grouped roots with diameters from 1.5 to 4.5 mm (Figure S1d). In the finest root class the percent root length was higher in MMS-1 than LHS-1, and higher with manure added than in pure simulants (Figure S1a), but differences between treatments were less pronounced than for absolute fine root length values shown in Figure 1c,d. Plants grown on LHS-1 allocated proportionally more root length to classes with diameters from 0.5 to 4 mm than those grown on MMS-1, with less root length percentage in simulants mixed with manure (Figure S1b–d). Roots with D > 4 mm were only found occasionally; therefore, data were highly variable and differences between treatments were not significant (Figure S1e). These roots represent a very small percentage in length (<0.5%) but a much higher percentage in weight and can account for part of the finding that root mass and length of MMS-1 were almost three-fold that of LHS-1, but root surface was only less than two-fold (Table 2), with the consequence that specific root surface was higher in LHS-1. In general, absolute values shown in Figure 1 and percentages shown in Figure S1 indicate a below-ground system made of thicker roots in LHS-1 than in MMS-1 and in pure simulants than in growth media with manure.

Manure amendment of MMS-1 and LHS-1 in mixtures used for this experiment resulted in a higher amount of nutrients, an increase in porosity and water retention, a reduction in bulk density, and a dilution of toxic substances found in pure simulants (Table S1—Supplementary Material and [28]). All of such improvements may be invoked to have an effect on our findings of larger, finer root systems in more productive treatments. A higher root length and finer root systems in plants are often interpreted in terms of response to a high level of N [22,36]; in lettuce enhanced root length, density at high N is reported [34,37]. Controversial behaviour is recorded for phosphorus: P deficiency is found to promote [38,39] or reduce [40] root proliferation, depending on species, but often results in finer root systems (e.g., [41,42]). In lettuce under low P, Beroueg et al. [40] reported a higher taproot growth with lower branching, although branch diameters were finer.

Lettuce was found to be very sensitive to compaction of the growth medium, even across narrow ranges of bulk density (1.25 to 1.50 g cm^−3^ [43]), partly overlapping with the wider range of bulk densities in our mixtures spanning from 1.390 to 0.812 in MMS-1 and from 1.792 to 0.869 g cm^−3^ in LHS-1.

Other relevant differences between Mars and Lunar pure simulants included a lower content of toxic elements and a higher CSC and content of some nutrients, porosity, and water holding for MMS-1 (Table S1—Supplementary Material and [28]). The LHS-1 simulant, though, was shown to have higher water holding than MMS-1 between suctions of 25 cm and 600 cm of an equivalent height of water, where the upper value is the matric potential at which lettuce water uptake starts slowing down due to water stress according to Taylor and Ashcroft [44]. This indicates a higher volume of readily available water for non-limited lettuce growth in LHS-1, which might be expected to reproduce effects of water availability reported in the literature on root proliferation and a higher proportion of fine roots [35]. However, in our case this potential superiority of LHS-1 was not large enough to offset the negative effects on fine root proliferation and overall growth, due to poorer ranking of the Lunar simulant compared to MMS-1 for other physical and all chemical properties (Table S1—Supplementary Material and [28]). We are unable to attribute final agronomic performance of growth media to any single factor among water availability, porosity, bulk density, concentrations of nutrients, and toxic elements, due to their contemporary variation and to interactive or offsetting effects. Interactions with management also add complexity to the comparison: the higher water retention between 25 and 600 cm would be meaningful only in case of low-frequency high-volume irrigation, whereas it would not give LHS-1 any particular advantage over MMS-1 in the case of high-frequency low-volume irrigation strategies, as the drip irrigation used in our experiment and other systems is likely to be used in space settings.

In our research the highest amendment rate (50:50, w:w) resulted in a reduction in plant above-ground performance in both simulants. This confirms findings of previous research [6]. Our data show a lower below-ground growth as well, and this cannot be directly related to nutrients or physical properties of growth media, except for a reduction in saturated hydraulic conductivity, which suggests macropore clogging by organic amendments and a possible impairment of aerobic processes.

**Figure 2:**
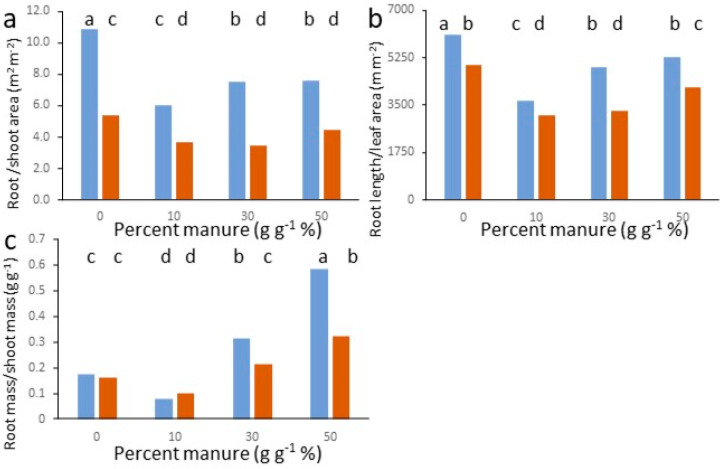
Interaction of simulant and manure concentration on allometric relations; (**a**) root-to-shoot mass ratio; (**b**) root-to-leaf surface area; and (**c**) root length per unit leaf area. Orange bars: MMS1; blue bars: LHS1. Bars with different letters are different for *p* < 0.05 at the post hoc Duncan’s mean separation test.

In our data root mass, surface, volume, total, and fine root length ranked close to plant leaf area ranking and indicate that maximum lettuce leaf production was obtained with a finer root system. Among allometric relation between above- and below-ground traits (Figure 2), the root-to-leaf area ratio was higher in plants grown on LHS-1 than on MMS-1, and in pure simulants compared to the corresponding amended treatments. No significant difference was found between manure concentrations within each simulant except for LHS-1, where values at 10% were lower than at 0, 30, and 50% manure. Similar trends were found for root length per unit leaf area (Figure 2b): they show that a higher investment in root surface or length is necessary to produce unit leaf area for plants grown on Lunar rather than Mars simulant at all manure levels, and that amendment increases root efficiency by decreasing root length to leaf area ratios. Root length per unit leaf area (Figure 2b), though, shows that lowest absolute values, corresponding to highest efficiency of roots, are found at 10% manure for both simulants, although for MMS1, values were not different from those at 30% manure.

Area ratios or the root length/leaf area ratio are a functional expression of the relative sizes of above- and below-ground exchange surfaces or their proxies [45], and in our case (Figure 2a,b), they provide a framework consistent with the functional equilibrium theory (e.g., [46]), where richer below-ground environments allow less investment in root systems per unit above-ground functional unit (e.g., leaf area). While our data indicate that the richer Mars simulant and amended treatments allow a more efficient crop production, though, there is no further decrease in unit root investment with increasing manure dose. In fact, the lowest below-ground unit investment corresponds to the 10% manure percentage (and 30% in MMS-1 as well). This is an indication of limiting conditions emerging at higher amendment doses, which limit efficiency and need to be investigated.

The root-to-shoot mass ratio (Figure 2c) shows a more hormetic [47] pattern than area ratios. Plants grown on LHS-1 had significantly higher ratios than on MMS-1 at manure concentrations of 30% and 50%, showing a proportionally higher investment in below-ground organs per unit above-ground mass produced. Values for both simulants were lowest at 10 % manure, with a mass investment in roots between 0.08 and 0.10 that of shoots, and highest at 50% manure where the ratio reached values between 0.50 and 0.60 in LHS-1 and around 0.30 in MMS-1. From the viewpoint of carbon partitioning our data represent below-ground C allocation ranging from about 8 to almost 60% of shoot mass. Values of 10 to 20% are common in the literature under different management systems (e.g., [32,34]). Values of 30% or higher—as found in our data at a manure concentration of 30 and 50%—are not uncommon in lettuce (e.g., [33]); nevertheless, they are considered high in view of resource optimisation for common terrestrial growth systems [32]. This indicates that maximum production in Mars and Lunar simulants is obtainable at around 30% manure, but with an excessive carbon cost, corresponding to inefficient allocation compared to a lower production at 10% manure. In addition to the functional balance between organs devoted to resource acquisition, the shoot/root mass ratio depends on many functions of roots and shoots, such as mechanical stability or transport; therefore, physiological balance is better judged based on area or area/length ratios [45,48]. The mass ratio, though, remains important for judging efficiency in allocation of assimilates, and especially so in space environments where inputs are scarce. Furthermore, in our case all allometric ratios (Figure 2a–c) indicate a lower efficiency of high manure rates compared to 10%. Agathokleou et al. [47] report that root/shoot mass ratio dose dependence in many instances follows a direct or inverse u-shaped relation as in our data; still an indication of higher production with lower efficiency needs optimisation of other management decisions or relief from constraints. In our growth media, we found an increase in total porosity with amendment (Table S1—Supplementary Material and [28]), but it is counterbalanced and overwhelmed by macropore clogging as manure content increases in the mixtures, with resulting reductions in saturated hydraulic conductivity (Ks). The maximum positive effect of manure is recorded for MMS-1 at 10% of manure content (Ks = 3.82 cm h^−1^) and for LHS-1 at 30% of manure content (Ks = 2.42 cm h^−1^). Mechanisms underlying high root/shoot ratios at high manure content linked to a reduction in macropore and Ks may be found in hormonal retardation of shoot stomatal behaviour and growth as reported when roots are exposed to consequences of waterlogging, such as low root zone temperatures [49] or poor soil aeration [36].

Interpretation of our results in view of space environment conditions would need a further level of complexity linked to reduced gravity effects on plant above- and below-ground behaviour. This is studied in spaceflight settings where weightlessness occurs, or commonly simulated through devices which compensate gravity with free fall forces or change the position of plants and thereby avoid constant gravity vectors [50]. Other artificial conditions are introduced by such tools [51], including magnetic or mechanical stress and breakage of large structures. Studies were conducted at the molecular, cell, and whole-plant morphology scale, at gravity levels close to those of the Moon (0.17 g) or Mars (0.38 g) (e.g., [52]). In such conditions, shoot phototropism was shown as enhanced in microgravity and at 0.1 g, while not at 0.3 g and at the control of 1 g, whereas root phototropism was enhanced in microgravity conditions only. This suggests that where gravity is not strong enough to orient plant growth, light may step in to do so. A series of processes and especially hormonal synthesis and relocation, as well as cell-wall modifications, were invoked to explain biometric and morphological changes of plants grown in microgravity, including reduced mass accumulation and organ size, as well as a higher root/shoot ratio and changes in internode length (e.g., for lettuce [53]), due to impaired balance between cell growth and proliferation [54]. Nevertheless Paul et al. [55] showed that even when plant and root size is smaller than that of ground-grown control plants, root growth away from shoots in response to directional light, and skewing and waving—which were considered gravity-dependent behaviours- are present in spaceflight microgravity conditions. Touch responses and auxins are invoked as alternative factors. Conservation of tropisms—the most studied phenomena in plant behaviour in space—at reduced gravity in spite of reduced plant size allows to comment that from the plant morphology point of view in first approximation, our results on plant growth media interactions may be relevant to the space environment even though our experiment was not conducted in microgravity conditions since root depth distribution and exploration of growth media would be reasonably conserved. Further steps, though, would involve research at reduced gravity in order to account for gravity effects on plant size and its implications for interactions with growth media.

### 2.2. Physiological Parameters of Lettuce Plants

Regardless of manure concentration, plants grown on Mars simulant exhibited a significantly higher SPAD index level (avg. 12.4) than Lunar substrate (avg. 10.5), while no significant differences were observed for fluorescence values (Table 3). However, the mean effect of amendment showed a significantly higher SPAD index value in the 30% treatment compared to pure simulants (13.4 and 9.2, respectively). In contrast, all manure applications recorded significantly higher fluorescence values (F_v_/F_m_) than the pure simulant. However, no significant interaction between the two tested factors was observed for both physiological parameters under investigation (Table 3).

The SPAD index is an effective non-destructive tool for an indirect measurement of chlorophyll content [56,57]. The dependence of photosynthesis on chlorophyll molecules as the primary medium of harvesting light energy to drive electron transport reactions was demonstrated [58]. In a similar experiment on lettuce grown at different mixtures of MMS-1 and green compost, photosynthetic rate decreased at higher compost concentrations [6]; this response was consistent with the reduction in SPAD index and dry biomass recorded in our work at the 50% manure dose. However, our highest biomass treatment (30% manure dose) showed 3-fold lower SPAD values than those recorded on butterhead lettuce grown with the nutrient film technique (NFT) under optimal environmental and nutrient availability conditions [59]. As well as the SPAD index, the maximum quantum efficiency of PSII (F_v_/F_m_) is also an indicator of photosynthetic efficiency and plant health [60,61]. Generally, F_v_/F_m_ values between 0.79 and 0.84 are approximate optimum values among unstressed leaves of many different species, while lower values indicate plant stress [62]. In our experiment, the very low fluorescence values recorded in plants grown on pure simulants indicated the occurrence of photosystem damage due to severe nutritional stress [63].

### 2.3. Extractable Nitrogen, Carbon, Microbial Biomass Nitrogen, and Carbon in Simulant/Manure Mixtures after Plant Growth

A significant increase in Extr. N and Extr. C in MMS-1 (78.0 mg kg^−1^) and LHS-1 (185.2 mg kg^−1^) simulants was observed, whereas the MBN and MBC values were greater in LHS-1 (92.0 and 523.2 mg kg^−1^, respectively) compared to the MMS-1 simulant (Table 4). By increasing the manure concentration, a significant increase in Extr. N, Extr. C, MBN, and MBC was observed. Furthermore, significant differences between *Rhizo* and *Bulk* samples were found only in Extr. N and MBN values (Table 4), and also by comparing the three interaction factors, such as simulant, amendment, and *Rhizo* vs. *Bulk* (*S × M × RB*; *p* < 0.001; Table 4). In particular, the value of Extr. N in MMS-1 was greater than that in LHS-1 (122.7 and 94.7 mg kg^−1^, respectively) within the *Bulk* samples amended with 30% manure. Conversely, the values of MBN in both *Rhizo* and *Bulk* samples of LHS-1 upon 30 and 50% manure treatment exceeded those of MMS-1 (Table 4).

No significant differences were found in terms of Extr. C and MBC by comparing *Rhizo* vs. *Bulk* and the three factors of interaction (*S × M × RB*) for Extr. C, whereas the interaction between simulants × amendment factors (*S × M*) highlighted always significant differences (Table 4).

Our results are consistent with literature reporting increases in MBC and MBN after organic amendment [18,64,65]. Zhang et al. [65] found a strong increase in MBC and MBN after horse manure-based amendment and they attributed this response to the readily metabolisable carbon and nitrogen in the applied manure. Additionally, Li et al. [64] observed an enhancement of MBC and MBN after 1 month from pig and cattle manure application in their field experiments carried on three different soils. Recently, Yu et al. [18] reported that pig manure had the best performance among different organic amendments in increasing soil MBC and MBN. In a soilless study, Sax and Scharenbroch [66] found wood chips or compost-based organic amendments of vermiculite, inert substrate used in growing nursery, as well as enhanced chemical and biochemical fertility, obtaining an increase in MBC, respiration, TOC, and TN.

### 2.4. Enzymatic Activities in Simulant/Manure Mixtures after Plant Growth

**Figure 3:**
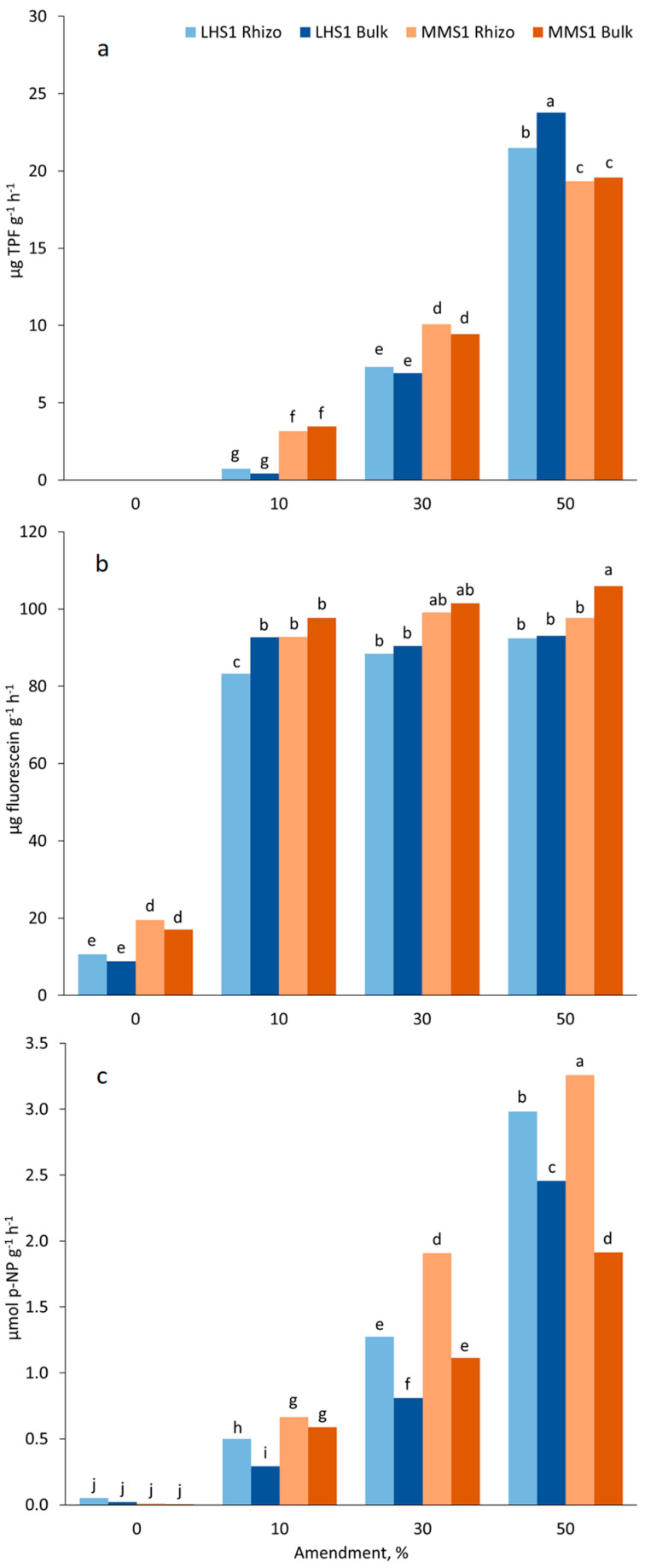
Interaction of simulant, manure concentration and *Rhizo* vs. *Bulk* soil on (**a**) dehydrogenase; (**b**) fluorescein diacetate hydrolysis; and (**c**) alkaline phosphomonoesterase activity. Bars with different letters are different for *p* < 0.05 at the post hoc Duncan’s mean separation test.

*Rhizo* and *Bulk* samples of LHS-1 and MMS-1 without manure amendment had no DH activity (Figure 3a). Upon manure addition DH activity increased with manure rate (10, 30, and 50%). At 10 and 30% manure, the DH activity was significantly greater in MMS-1 and no significant differences between *Rhizo* and *Bulk* samples were observed. At 50% manure concentration, the DH activity grew more in LHS-1 compared to MMS-1, reaching 21.5 and 23.8 μg TPF g^−1^ h^−1^ in *Rhizo* and *Bulk* samples, respectively (Figure 3a).

Dehydrogenases are intracellular enzymes involved in redox processes of a wide range of organic molecules and their activity is related to living microbial organisms [67]. DH activity is strictly correlated with soil microbial biomass and its metabolic activity [68]. The activity of these enzymes solely is greater in the rhizosphere because of the presence of the root–microorganism system in which a greater abundance of microorganisms occurs [69]. In our experiment, there were no significant differences between *Rhizo* and *Bulk* soils by increasing manure rates until 50%.

Most of samples *Rhizo* and *Bulk* MMS-1 and LHS-1 exceeded 90 μg fluorescein g^−1^ h^−1^ (Figure 3b) already at a 10% manure rate according to the findings of Bonanomi et al. [70], who found an enhancement in the FDA activity upon organic amendments in soil. Although in the literature no significant differences were registered between rhizospheric and non-rhizospheric media after compost amendment [71], *Rhizo* LHS-1 at a 10% rate and *Rhizo* MMS-1 at a 50% rate of manure showed a slightly reduced FDA activity in respect to *Bulk* soil (Figure 3b). At manure doses higher than 10%, no further stimulation of FDA activity occurred, and at 30 and 50% manure, MMS1 *Bulk* samples showed greater activity levels than LHS-1 samples. At 50% manure addition, the *Bulk* sample MMS1 reached the greatest FDA activity level (105.9 μg fluorescein g^−1^ h^−1^; Figure 3b). Pure simulants showed FDA activity anyway, although it was small (Figure 3b) due to microorganisms whose presence is demonstrated by the MBC data (Table 4). The greater FDA activity recorded in MMS-1 could be explained by a more intense rhizosphere effect since lettuce plants grew up better in MMS-1, as with all biometric parameters highlighted (Table 1 and Table 2).

Pure LHS-1 and MMS-1 simulants had an almost zero AP activity (Figure 3c). Values of AP activity increased with manure percentage in the mixtures (Figure 3c) and this is in agreement with Yang et al. [72], Gupta et al. [73], and Liu et al. [74]. In general values, *Rhizo* samples were higher than *Bulk* ones and reached 3.3 μmol p-NP g^−1^ h^−1^ at 50% manure rate in MMS-1 (Figure 3c), in coincidence with P demand of plant and microorganisms that could stimulate this enzyme activity [72]. Zymography studies, based on a peculiar technique to visualise the spatial distribution of potentially active enzymes in soil with 2D images, highlighted an intense phosphatase activity close to the roots [75,76,77]. Phosphatase activity is generally higher in the rhizosphere compared to bulk soil, as this enzyme is either directly released by roots or by microorganisms that are stimulated by rhizodeposits [78]. Spohn and Kuzyakov [79] evaluated alkaline and acid phosphatase near the lupine root, and they found the alkaline phosphatase in *Rhizo* was up to 5.4 times greater than in *Bulk* soil.

### 2.5. Nutrient Bioavailability in Simulant/Manure Mixtures after Plant Growth

The concentration of the main macro and micronutrients in different MMS-1 or LHS-1/manure mixtures (separated in *Rhizo* and *Bulk* soil after lettuce growth), extracted by 1 M NH_4_NO_3_ to assess the promptly bioavailable fractions (BS ISO 19730, 2008) and 0.05 M EDTA at pH 7 to evaluate the potentially bioavailable fractions [80], is shown in Table 5 and Table S2 (expressed in mg kg^−1^ DW) and Tables S3 and S4 of Supplementary Material (expressed as % of the total content of each nutrient).

The promptly (Table 5 and Table S3) and potentially (Tables S2 and S4) bioavailable fractions of Ca, K, Mg, P, and Mn extracted from MMS-1-containing mixtures were significantly higher than those extracted from LHS-1-based mixtures, while the opposite was observed with Fe and Na (and promptly bioavailable Cu and Zn). In most of the cases, this trend was also recognised at the start point before lettuce growth, and it is mainly due to the higher total nutrient contents in the MMS-1- than LHS-1-based mixtures (Table S1—Supplementary Material and [28]). Despite mixtures with LHS-1 containing more Ca than Mars simulant-based substrates, they are a lower source of promptly and potentially bioavailable Ca for plants and rhizosphere biota; in contrast, they released larger amounts of promptly and potentially bioavailable Na in comparison to MMS-1-containing mixtures, and this can also explain the different alkalinity and chemical properties of the two simulants. As recently discussed by Duri et al. [9] in their review on the potential for Lunar and Martian regolith simulants to sustain plant growth, plants take up only the bioavailable forms of nutrients from a simulant-based growth substrate, not the elements occluded in mineral structures that are released only after mineral weathering. Hence, plants can exploit only a low-to-moderate fraction of the total nutrient contents in a simulant to satisfy their requirements.

The amendment of MMS-1 and LHS-1 simulants with increasing rates of monogastric-based manure determined a significant increase in the promptly (Table 5 and Table S3) and potentially (Tables S2 and S4 of Supplementary Material) bioavailable fractions of the macro and micronutrients. Specifically, the nutrient bioavailable fractions in the 90:10, 70:30, and 50:50 simulant/manure mixtures were, respectively, 11-, 24-, and 32-fold (Table 5), and 5-, 11-, and 14-fold (Table S2—Supplementary Material), higher than those in the pure simulants (100:0). Likewise, in comparison to pure MMS-1 simulant, Caporale et al. [5] noted an increase in a water-soluble fraction of nutrients, such as Ca, K, Mg, nitrate, phosphate, and sulphate, when they amended the simulant with green compost at increasing rates (up to 70% of compost in volume).

For the majority of the nutrients, no statistically significant differences between promptly and potentially bioavailable fractions extracted from *Rhizo* soil and those extracted from *Bulk* soil were found. Actually, except for 100:0 treatment, the substrate separation into *Rhizo* vs. *Bulk* soil was very challenging and purely indicative, due to the abundance of root biomass into a relatively small volume of each pot. Nevertheless, a significant depletion of promptly and potentially bioavailable K and Mg in the *Rhizo* vs. *Bulk* soil occurred, probably due to a fast uptake rate of two macronutrients by the lettuce plants in the last growth phase. In contrast, there was a significant increase in the promptly bioavailable Cu in the *Rhizo* vs. *Bulk* soil, maybe due to release of root exudates, rhizosphere pH acidification, and enhanced microorganism activity. The interaction among the three factors: simulants (S) × amendment (M) × *Rhizo*/*Bulk* soil (RB), was significant (*p* < 0.05) only for the promptly bioavailable Mn (Table 5), and not significant in all the other cases. On the other hand, the interaction between simulants (S) × amendment (M) factors was significant for the majority of nutrients, except promptly and potentially bioavailable K and Zn (Table 5 and Table S2—Supplementary Material).

The monitoring of the promptly and potentially bioavailable fractions of nutrients in the simulant/manure mixtures, before (Table S1—Supplementary Material and [28]) and after the lettuce growth cycle (Table 5 and Table S2—Supplementary Material), evidenced an overall reduction in potentially bioavailable fractions of the macro and micronutrients, mainly due to plant uptake and bioaccumulation in microbial biomass.

The release and mobilisation of these nutrients from mineral and organic moieties of substrates, regulated by the intense root and microbial activity and enhanced by water periodic supply, induced an increase in the promptly bioavailable pool of Ca, Mg, and Na at the end of plant growth, in comparison to the start point described by Caporale et al. [28]. Indeed, at least for Ca, this phenomenon may be also due to the release of nuclear Ca^2+^ by plant root, which is essential to the modulation of the plant growth hormone auxin and establishment of nitrogen-fixing and phosphate-delivering arbuscular mycorrhizal endosymbiosis [81]. Unlike Ca and Mg (whose promptly bioavailable pool raised up to 58%), the promptly bioavailable fraction of Na at the end of plant growth was on average 5-fold and 7-fold higher than the start point, respectively, in MMS-1 or LHS-1/manure mixtures (100:0 excluded). This abundance of promptly bioavailable Na potentially caused a salt stress in plants [5,6,82], which could justify, at least in part, the lower growth and agronomic performance of lettuces grown on LHS-1-based vs. MMS-1-based substrates.

Since the bioavailability of nutrients in plant growth media is governed by the pseudo-equilibrium between aqueous and solid phases, the physico-hydraulic properties of the different mixtures, assessed by Caporale et al. [28], played a key role. Nevertheless, for reliable future applications, these features need to be better studied in microgravity conditions. Indeed, by comparing the hydraulic properties of selected media measured both on Earth and in microgravity, narrowed pore size distributions were highlighted [83]; moreover, the large pores were basically inactive in microgravity conditions. This evidence allows us to argue that water availability in our simulant/manure mixtures will be lower than that calculated under terrestrial conditions.

Further, the absence of the gravitational field can lead to a reduction in water circulation into the growing medium; hence waterlogging in the root zone due to inadequate moisture distribution in the root substrate can cause stress in microgravity conditions [84,85] and then lower the nutrient bioavailability [28].

From the results of experiments made aboard the International Space Station [85], an apparent reduction in mean volume diffusive transport in microgravity conditions was found. This could increase the propensity for anoxia, especially for finer regolith media.

### 2.6. Leaf Mineral Content and Plant Nutrients Uptake

The mean effect of amendment showed a significant increase of about 12- and 2-fold in phosphate and Mg concentration, respectively, at the highest manure dose compared to the pure simulant (Table 6). Potassium, Ca, Na, and SO_4_ contents incurred significant interaction of the tested factors (S × M) (Table 6). In particular, the Martian mixtures showed, at the highest manure dose, an increase in K, Ca, and SO_4_ content by 162%, 154%, and 600%, respectively, compared to the pure MMS-1 simulant, while Na content was significantly higher at doses 30 and 50%. Regarding plants grown on Lunar simulant, K and SO_4_ concentration was, on average, 70% and 248% higher in manure-treated plants than in the untreated substrate, while Na content at the 50% manure dose was significantly higher compared to all other treatments. In contrast, the Ca content in the Lunar mixtures was significantly higher: 96% at the 30% manure dose compared to the pure simulant (Table 6).

Regardless of amendment factor, the mean effect of the simulant shows significantly higher uptake of all analysed elements in plants grown on MMS-1 (Table S5—Supplementary Material). In turn, the plant uptake of all elements analysed was affected by the S × M significant interaction. In Martian mixtures, per-plant uptake of PO_4_, Mg, Ca, and Na was significantly higher at the 30% manure dose (109-, 23-, 13-, and 23-fold more than pure simulant, respectively), whereas the amount of K and SO_4_ assimilated per plant was significantly higher at the 30% and 50% manure doses (on average, 18- and 49-fold more than pure simulant, respectively). Regarding the Lunar simulant, with the exception of Na, whose highest values were recorded in all manure-treated mixtures, PO_4_, K, Mg, Ca, and SO_4_ uptake was significantly higher in plants grown on the 10% and 30% manure-treated mixtures (on average 66-, 14-, 9-, 14-, and 26-fold more than pure simulant, respectively) (Table S5—Supplementary Material).

Trends in leaf mineral content were consistent with those on nutrient bioavailability discussed in the previous section. The increase in nutrient bioavailability in the growth media with organic matter amendment was widely demonstrated [86,87]. In our work, PO_4_, K, and Mg increased linearly in both simulant mixtures and leaf tissues when the dose of manure increased. Specifically, bioavailable Na levels increased more than proportionally in both simulant/manure mixtures, reaching significantly higher contents in LHS-1-based substrates than in MMS-1 ones (Table 5 and Table S2—Supplementary Material); this finding reflected the notably high Na concentration found in lettuce leaves at the highest manure dose. The latter result may explain the reduction in dry biomass recorded at the 50% manure dose due to the detrimental effects of Na, as reported in several other works on lettuce [88,89]. In addition, as demonstrated in the literature [90,91], the high Na content found in LHS-1 at the highest manure dose also resulted in reduced Ca assimilation, further contributing to the severe reduction in dry biomass recorded in plants grown in this simulant mixture.

Overall, the assessment of the leaf biomass nutritional status demonstrated that lettuce can be a long-term dietary source of mineral nutrients for space crews. The nutritional and nutraceutical qualities of these plants were also evaluated elsewhere [92], through the analysis of bioactive compounds (i.e., content of organic acids and carotenoids, and phenolic profile) and the assessment of antioxidant activity (ABTS and DPPH assays).

## 3. Materials and Methods

### 3.1. Main Mineralogical, Physico-Hydraulic, and Chemical Properties of MMS-1 and LHS-1 Simulants, Horse/Swine Monogastric Manure, and Related Simulant/Manure Mixtures

A summary of the mineralogical and elemental composition and the main physico-hydraulic and chemical properties of MMS-1 and LHS-1 simulants, horse/swine monogastric manure, and related simulant/manure mixtures, is provided in Table S1 of Supplementary Material, gathered from and widely discussed in Caporale et al. [28]. Briefly, both simulants are alkaline and coarse textured with low water holding capacity. The LHS-1 Lunar Highlands simulant (Exolith Lab, Center for Lunar and Asteroid Surface Science of University of Central Florida, Orlando, FL, USA) comprises abundant plagioclases and short-range-order minerals, with little amounts of phyllosilicates (chlorite and kaolinite). In comparison with MMS-1, LHS-1 simulant shows lower bioavailability of nutrients, total porosity, saturated hydraulic conductivity, and water retention, but higher bioavailability of potentially toxic elements. The MMS-1 Mojave Mars simulant (The Martian Garden, Austin, TX, USA) is made mainly of plagioclase, amorphous minerals and zeolite, and secondary of hematite and smectite; it can be a potential source of bioavailable Ca, Mg, and K for plant growth, but at the same time, it shows a series of physicochemical properties negatively impacting plant growth [6]. The horse/swine monogastric manure (Jolly Pellet, Agraria Di Vita srl, Pistoia, Italy) is characterised by a low C/N ratio, hence it can provide a significant amount of potentially available N for rhizosphere microorganisms and plant roots. At the same time, according to the medium-low H/C value, it comprises a significant aromatic moiety as well, ensuring a good stability of the organic matter over time. It is also an important source of nutrients; however, it contains a significant amount of Na, which negatively raises its pH and electrical conductivity. The mix of the poorly fertile MMS-1 or LHS-1 simulants with increasing rates of monogastric-based manure (i.e., 100:0, 90:10, 70:30, 50:50, *w*/*w*), improved significantly the physical, chemical, and biological fertility of the substrates, providing energy and essential nutrients for rhizosphere activity, and colloidal and chemically reactive compounds promoting particle aggregation and formation of water-retaining microporosity. MMS-1/manure mixtures have a better chemical fertility (lower pHs and higher nutrient availability) than LHS-1/manure ones; this divergent fertility was particularly evident at 90:10 w:w rate and tended to be mitigated by increasing the levels of manure. On the other hand, LHS-1/manure mixtures exhibit a better water retention than MMS-1/manure ones, especially in the ‘dry’ region of matrix potential head (between −100 and −600 cm).

### 3.2. Plant Material, Growth Chamber Condition, and Experimental Treatments

Lettuce seedlings (*Lactuca sativa* L. cultivar ‘Grand Rapids’, West Coast Seeds, Vancouver, Canada) were grown in the nursery using polystyrene trays filled with vermiculite. At the third true leaf stage, plants were transplanted into plastic pots (9 × 9 × 9 cm) filled with different mixtures of simulants and monogastric manure and transferred to the growth chamber. Experimental treatments consisted of two simulants, Mars MMS-1 and Lunar LHS-1, mixed at different rates (100:0, 90:10, 70:30, and 50:50, w:w) with ground horse/swine monogastric manure (sieved to 2 mm).

The experiment was carried out at the experimental farm of the Department of Agricultural Sciences, University of Naples Federico II (Italy) in a walk-in open gas exchange climate chamber (28 m^2^: 7.0 × 2.1 × 4.0 m; W × H × D). HPS lamps (Master SON-T PIA Plus 400 W, Philips, Eindhoven, The Netherlands) were used to provide 400 µmol m^−2^ s^−1^ light intensity at canopy level with a 16/8 h photoperiod (light/dark) under ambient CO_2_ concentration conditions (370–410 ppm). A day/night air temperature and humidity regime of 22/18 °C and 60/80%, respectively, was provided through two heating, ventilation, and air conditioning (HVAC) systems and a fog system. Plants were irrigated throughout the crop cycle (30 days from transplanting to harvest) with only osmotised water using a drip irrigation system (open loop) equipped with 2 L h^−1^ self-compensating drippers.

### 3.3. Morpho and Physiological Measurements

One day before harvest, (30 days after transplanting; DAT) the maximum plant height (H) and average diameter (D_m_; as the average of two transverse diameters, where one of the two was the maximum diameter) of canopy of all plants were measured and then used to determine the growth index (GI) by the formula [3.14 · (D_m_/2)^2^ ·H]. At the same time, SPAD index and chlorophyll fluorescence were measured using a portable chlorophyll meter (SPAD-502, Minolta Corp. Ltd., Osaka, Japan) and a portable fluorometer (F_v_/F_m_ Meter, Opti-Sciences Inc., Hudson, NH, USA), respectively. According to Kitajima and Butler [93] the maximum efficiency of photosystem II (PSII) was calculated as F_v_/F_m_, with F_v_ = F_m_ − F_0_. In particular, F_0_, the ground fluorescence signal, was induced on 10 min dark-adapted leaves by a blue LED internal light of 1–2 µmol m^−2^ s^−1^ and F_m_, and the maximal fluorescence was induced by 1 s of saturating light pulse of 3000 µmol m^−2^ s^−1^.

At harvest, plants were cut at the soil level and shoot fresh weight (g plant^−1^) and number of leaves (LN) per plant was recorded, while leaf area (LA, cm^2^ plant^−1^) was measured using an electronic area meter (LI-COR 3100C Biosciences, Lincoln, NE, USA). Harvested tissues were oven-dried at 70 °C to constant weight (~72 h) for the determination of dry weight (dw, g plant^−1^) and leaf dry matter fraction (DM, %).

After cutting the above-ground part, pots were turned on the side and their content was gently extruded. Roots were brushed free of soil and then washed on a 0.5 mm sieve. All soil was then submerged with water, in order to collect remaining root fragments by elutriation.

Image analysis of roots was performed with the WinRhizo ArabidopsisV2009c (Regent Instruments Inc., Chemin Sainte-Foy, Québec, Canada) image analysis software on root systems placed in a 20 × 25 cm transparent tray with a 5 mm deep layer of water and scanned at 600 dpi by STD4800 Image Acquisition System. The following traits were measured: root surface area (RA, cm^2^ plant^−1^), mean diameter (D, mm), root volume (RV, cm^3^ plant^−1^), total root length (RL, m plant^−1^), and length separated in 10 diameter classes (from 0.0 to >4.5 mm in increments of 0.5 mm).

After scanning, roots were oven dried at 70 °C until constant weight and weighed to obtain the root dry mass (Rdw, g plant^−1^).

The specific root surface (SRS, m^2^ g^−1^) was calculated as the ratio of total surface to total length of roots. Indices of allometric relations between above- and below-ground plant parts were calculated as root-to-shoot biomass ratio (RSw, g g^−1^), and root-to-leaf area ratio (RLA, m g^−1^).

### 3.4. Determination of Microbial Biomass Carbon and Nitrogen in Manure Amended Simulants

Microbial biomass carbon (MBC) was measured according to the fumigation extraction method [67,94]. Briefly, 10 g of moist simulant were exposed to CHCl_3_ for 24 h at 25 °C and then treated with 40 mL of 0.5 M K_2_SO_4_ for 30 min under orbital shaking at 200 rpm; the suspension was filtered through Whatman 42 filter paper. A non-fumigated control underwent the same procedures described above without the CHCl_3_ exposure. Organic carbon in the extracts was determined after oxidation with 0.033 M K_2_Cr_2_O_7_ at 110 °C for 1.5 h by titration with 0.1 M Mohr salt solution. Results are expressed in mg C kg^−1^ dried samples.

Microbial biomass nitrogen (MBN) was measured on 0.5 M K_2_SO_4_ extracts according to Brookes et al. [95] and by the alkaline persulfate oxidation [96], with some modification. Briefly, an aliquot of the extract (1 mL) was diluted to 20 mL with deionised water in falcon tubes and 20 mL of the oxidizing reagent were added. The tubes were placed in an autoclave for 30 min at 120 °C. After that, the tubes were left to cool at room temperature and nitrate was determined by a UV spectrophotometer (PerkinElmer, UV/Vis Lambda 365) at 220 nm. MBN was calculated using a K_EN_ factor of 0.54 [95]. The total nitrogen concentration was calculated on calibration curve by using glycine at different concentrations of N (0.1, 0.2, 0.4, 0.6, 1, 1.5, 2.5, 5, 7, 10 mg L^−1^). Results are expressed in mg N kg^−1^ dried samples. All determinations were in triplicate.

### 3.5. Enzymatic Activity Assay in Manure Amended Simulants

Enzyme activities were determined within 15–20 d from the collection of the simulant samples stored at 4 °C. Dehydrogenase (DH) was determined with tetrazolium salts (TTC) solution as described by Alef and Nannipieri [97]. The fluorescein diacetate hydrolysis (FDA) was assessed as described by Green et al. [98]. Alkaline phosphomonoesterase (PHO) was determined according to Tabatabai and Bremner [99]. Triplicates were analysed for each activity assay.

### 3.6. Nutrient Bioavailability in Simulant/Manure Mixtures after Plant Growth

Promptly (i.e., readily soluble) and potentially bioavailable fractions of the main macro and micronutrients were extracted in triplicate from simulant/manure mixtures after the plant growth cycle by 1 M NH_4_NO_3_ (solid/solution ratio: 1/25; reaction time: 2 h; BS ISO 19730, 2008) and 0.05 M EDTA at pH 7 (solid/solution ratio: 1/10; reaction time: 1 h; [80]), respectively; the extracts were then filtered through filter papers (Whatman 42) and analysed by inductively coupled plasma—optical emission spectrometry (ICP-OES, Thermo Scientific iCAP 7400, Waltham, MA, USA).

### 3.7. Mineral Analysis and Calculation of Plant Nutrients Uptake

A subsample of dried leaves was ground and sieved to 0.5 mm using a cutting-grinding head mill (MF 10.1, IKA, Staufen im Breisgau, Baden-Württemberg, Germany) for determination of water-extractable cationic (Ca, K, Mg) and anionic (phosphate: PO_4_; sulphate: SO_4_) nutrient contents in leaves, according to the method described by Pannico et al. [100]. Briefly, 250 mg of dried samples were extracted in 50 mL of ultrapure water, incubated at 80 °C in a shaking water bath (ShakeTemp SW22, Julabo, Seelbach, Germany) for 10 min and then filtered by a nylon syringe filter with a 0.45 µm pore size (Phenomenex, Torrance, CA, USA). The content of anions and cations was detected by ion chromatography (ICS-3000, Dionex, CA, USA) coupled to an electrical conductivity detector. Plant nutrients uptake (mg plant^−1^) of each element was calculated using the following formula: dry plant biomass (g plant^−1^ dw) × element concentration (mg g^−1^ dw).

### 3.8. Statistical Analysis

The experimental design consisted of a factorial combination of the two simulants and four different substrate mixtures for a total of eight treatments with three replicates. A randomised complete block design was adopted, with a total of 16 experimental units of six plants each (for total of 96 plants). For soil nutrients and enzymatic activity, a third experimental factor was introduced since at the end of plant growth, the soil was collected in two fractions: *Rhizo*; the soil retained around roots after gently shaking, and *Bulk*; the rest of the soil.

The analysis of variance was therefore conducted as two-way or three-way ANOVA using the software package IBM SPSS Statistics v26 (SPSS Inc., Chicago, IL, USA). When separation of means was required, it was conducted through Duncan’s multiple range test (DMRT), performed at *p* ≤ 0.05. Relations between selected traits were analysed through correlation or regression analysis.

## 4. Conclusions

This study demonstrated that pure MMS-1 Mars and LHS-1 Lunar simulants can sustain plant growth (at least leafy vegetables such as lettuce), even in the absence of fertilisation. These simulants (i.e., an assemblage of terrestrial crushed rocks build up to replicate the physicochemical properties of extra-terrestrial regoliths, assessed in situ by previous missions), however, hold several properties that can hinder plant growth, such as alkaline pH, low content of promptly bioavailable nutrients but high Na bioavailability, predominance of macro vs. micropores and consequent scant water holding capacity, etc. The amendment of these nutrient-poor and alkaline substrates with stabilised organic matter, such as horse/swine monogastric manure at varying rates (100:0, 90:10, 70:30, 50:50, *w*/*w*), mitigated these negative features and significantly improved the ability of MMS-1 and LHS-1 simulants to sustain plant growth, even through the enhancement of microbial biomass abundance and activity. The mixture containing 70% in weight of simulant and 30% of manure provided the best outcomes in terms of biomass production and plant vigour/health. Additionally, this mixture is a more sustainable option for a BLSS developed with ISRU strategy than 50:50 simulant/manure growth medium. However, to assess the feasibility of these manure-amended Lunar and Martian soils for plant growth in space settings, these findings need to be validated in follow-up experiments under microgravity. In this context, the diverse water movement and dynamics in the soil/plant system can differently regulate the extent of mineral weathering and the rate of organic matter decomposition, the biogeochemistry and bioavailability of nutrients, and consequently, plant growth, physiology, and health. Moreover, the monitoring of fertility, properties, and terraforming processes occurring in the manure-amended Lunar and Martian soils over time, under consecutive cultivation cycles of different crop species, is of paramount importance to widen scientific knowledge in sustainable space food production systems.

## Figures and Tables

**Table 1:** Foliar biometric parameters in different mixtures of MMS-1 or LHS-1 simulants and manure (simulant/manure rates: 100:0, 90:10, 70:30, 50:50; *w*/*w*).

Source of Variance	Growth Index	Leaf Number	Leaf Area	Dry Biomass	Dry Matter
		No. plant^−1^	cm^2^	g plant^−1^	%
*Simulants (S)*					
MMS1	1458 ± 266	13.75 ± 1.14	450 ± 79.1	4.33 ± 0.75	16.1 ± 1.77
LHS1	512 ± 109	8.91 ± 0.62	127 ± 28.1	1.38 ± 0.32	18.2 ± 2.19
	***	***	***	***	***
*Amendment (%) (M)*					
0	31.8 ± 6.09 c	6.69 ± 0.44 d	16.8 ± 4.50 d	0.43 ± 0.07 c	27.7 ± 1.13 a
10	1153 ± 208 b	13.29 ± 1.25 b	355 ± 74.2 b	4.34 ± 0.81 a	16.9 ± 0.63 b
30	1645 ± 311 a	14.08 ± 1.47 a	490 ± 113 a	4.56 ± 1.05 a	13.3 ± 0.54 c
50	1109 ± 352 b	11.25 ± 1.28 c	292 ± 99.0 c	2.09 ± 0.76 b	10.7 ± 0.48 d
	***	***	***	***	***
*S* × *M*					
MMS1 × 0	45.0 ± 2.78 de	7.56 ± 0.29 e	26.5 ± 2.7 f	0.58 ± 0.07 e	25.3 ± 0.17 b
MMS1 × 10	1588 ± 153 b	16.00 ± 0.67 b	521 ± 14.2 b	6.10 ± 0.30 b	16.5 ± 0.32 c
MMS1 × 30	2332 ± 105 a	17.33 ± 0.19 a	740 ± 23.7 a	6.88 ± 0.32 a	12.8 ± 0.76 de
MMS1 × 50	1866 ± 218 b	14.11 ± 0.11 c	512 ± 24.1 b	3.77 ± 0.25 c	9.77 ± 0.29 f
LHS1 × 0	18.6 ± 1.80 e	5.83 ± 0.36 f	7.2 ± 0.2 f	0.29 ± 0.02 e	30.1 ± 0.80 a
LHS1 × 10	717 ± 56.2 c	10.58 ± 0.22 d	190 ± 7.2 d	2.58 ± 0.29 d	17.2 ± 1.33 c
LHS1 × 30	958 ± 44.3 c	10.83 ± 0.44 d	240 ± 17.6 c	2.23 ± 0.20 d	13.9 ± 0.76 d
LHS1 × 50	353 ± 39.0 d	8.39 ± 0.20 e	71.8 ± 4.7 e	0.40 ± 0.02 e	11.6 ± 0.48 ef
	***	***	***	***	*

Non-significant (ns). *, *** significant at *p* ≤ 0.05 and 0.001, respectively. Simulants (S), amendment (M), and Rhizo vs. bulk soil (RB) and interaction were compared by Duncan’s multiple range test (*p* = 0.05). Different lowercase letters within each column indicate significant differences (*p* ≤ 0.05).

**Table 2:** Root biometric parameters in different mixtures of MMS-1 or LHS-1 simulants and manure (simulant/manure rates: 100:0, 90:10, 70:30, 50:50; *w*/*w*).

Source of Variance	Root Dry Mass	Root Length	Root Surface Area	Root Volume	Specific Root Surface
	g plant^−1^	m plant^−1^	cm^2^ 10^−2^ plant^−1^	cm^3^ plant^−1^	m^2^ g^−1^
*Simulants (S)*					
MMS1	0.84 ± 0.18	158.52± 29.92	17.28 ± 3.11	15.2 ± 2.53	0.23 ± 0.02
LHS1	0.30 ± 0.08	57.21 ± 13.70	8.93 ± 2.23	11.3 ± 2.80	0.30 ± 0.05
	*	**	*	n.s.	n.s.
*Amendment (%) (M)*					
0	0.07 ± 0.01 d	8.74 ± 1.47 c	1.39 ± 0.24 c	1.85 ± 0.34 d	0.18 ± 0.02 b
10	0.40 ± 0.06 c	116.26 ± 15.45 b	15.11 ± 1.49 b	16.28 ± 1.51 b	0.43 ± 0.04 a
30	1.08 ± 0.13 a	180.67 ± 22.67 a	21.81 ± 1.71 a	21.99 ± 1.47 a	0.23 ± 0.02 b
50	0.71 ± 0.2 b	125.78 ±33.17 b	14.11 ± 3.32 b	12.87 ± 2.57 c	0.22 ± 0.01 b
	**	**	**	**	**
*S* × *M*					
MMS1 × 0	0.09 ± 0.01 e	13.15 ± 0.70 f	2.01 ± 0.22 d	2.55 ± 0.40 de	0.21 ± 0.02 c
MMS1 × 10	0.60 ± 0.01 c	163.24 ± 2.23 c	18.88 ± 0.51 b	17.62 ± 0.98 bc	0.32 ± 0.01. b
MMS1 × 30	1.46 ± 0.01 a	243.83 ± 12.90 a	25.44 ± 1.02 a	21.28 ± 0.64 a	0.17 ± 0.03 d
MMS1 × 50	1.18 ± 0.21 b	213.8 ±28.38 b	22.79 ± 2.96 a	19.3 ± 2.45 b	0.21 ±0.01 c
LHS1 × 0	0.05 ± 0.00 e	4.33 ± 0.30 f	0.77 ± 0.04 e	1.17 ± 0.09 e	0.16 ± 0.01 d
LHS1 × 10	0.21 ± 0.21 d	69.29 ± 6.24 e	11.35 ± 1.32 c	14.94 ± 2.09 c	0.54 ± 0.01 a
LHS1 × 30	0.69 ± 0.06 c	117.51 ± 10.94 d	18.18± 1.73 b	22.70 ± 2.20 a	0.28 ± 0.03 bc
LHS1 × 50	0.24 ± 0.02 d	37.71 ± 1.34. f	5.44 ± 0.09 d	6.39 ± 0.04 d	0.24 ± 0.01 c
	**	**	**	**	**

Non-significant (ns). *, ** significant at *p* ≤ 0.05 and 0.01, respectively. Simulants (S), amendment (M), and Rhizo vs. bulk soil (RB) and interaction were compared by Duncan’s multiple range test (*p* = 0.05). Different lowercase letters within each column indicate significant differences (*p* ≤ 0.05).

**Table 3:** SPAD index and fluorescence in different mixtures of MMS-1 or LHS-1 simulants and manure (simulant/manure rates: 100:0, 90:10, 70:30, 50:50; *w*/*w*).

Source of Variance	SPAD Index	Fluorescence
		F_v_/F_m_ Ratio
*Simulants (S)*		
MMS1	12.40 ± 0.53	0.738 ± 0.02
LHS1	10.51 ± 0.57	0.721 ± 0.03
	***	ns
*Amendment (%) (M)*		
0	9.27 ± 0.57 c	0.579 ± 0.04 b
10	11.63 ± 0.35 b	0.756 ± 0.01 a
30	13.48 ± 0.53 a	0.803 ± 0.00 a
50	11.42 ± 0.93 b	0.781 ± 0.01 a
	***	***
*S × M*		
MMS1 *×* 0	10.12 ± 0.59	0.612 ± 0.03
MMS1 *×* 10	11.79 ± 0.71	0.744 ± 0.00
MMS1 *×* 30	14.31 ± 0.48	0.800 ± 0.01
MMS1 *×* 50	13.36 ± 0.10	0.798 ± 0.01
LHS1 *×* 0	8.41 ± 0.75	0.547 ± 0.08
LHS1 *×* 10	11.46 ± 0.30	0.767 ± 0.00
LHS1 *×* 30	12.66 ± 0.70	0.806 ± 0.00
LHS1 *×* 50	9.49 ± 0.75	0.765 ± 0.01
	ns	ns

Non-significant (ns). *** Significant at *p* ≤ 0.001. Simulants (S), amendment (M), and Rhizo vs. bulk soil (RB) and interaction were compared by Duncan’s multiple range test (*p* = 0.05). Different lowercase letters within each column indicate significant differences (*p* ≤ 0.05).

**Table 4:** Extractable nitrogen (Extr. N) and carbon (Extr. C), microbial biomass nitrogen (MBN), and carbon (MBC) in different mixtures of MMS-1 or LHS-1 simulants and manure (simulant/manure rates: 100:0, 90:10, 70:30, 50:50; *w*/*w* %), separated in rhizo and bulk soil after lettuce growth.

Source of Variance	Extr. N	Extr. C	MBN	MBC
	mg kg^−1^ DW			
*Simulants (S)*				
MMS1	78.0 ± 56.4	152.6 ± 101.0	77.8 ± 50.3	397.4 ± 350.2
LHS1	75.0 ± 52.7	185.2 ± 120.2	92.0 ± 67.6	523.2 ± 548.4
	***	***	***	***
*Amendment % (M)*				
0	11.6 ± 2.9 d	32.5 ± 10.6 d	11.4 ± 3.5 d	46.2 ± 17.2 d
10	39.9 ± 4.3 c	111.3 ± 15.0 c	57.5 ± 9.9 c	152.6 ± 42.7 c
30	112.6 ± 11.8 b	218.6 ± 48.9 b	110.4 ± 34 b	500.6 ± 152.3 b
50	141.7 ± 20.9 a	313.1 ± 40.0 a	160.2 ± 20.9 a	1141.8 ± 304.1 a
	***	***	***	***
*Rhizo* vs. *bulk soil (RB)*				
RH	81.7 ± 60.9	166.4 ± 110.5	94.9 ± 66.4	450.6 ± 427.6
BK	71.3 ± 47.0	171.4 ± 113.9	74.8 ± 50.9	470.0 ± 498.4
	***	ns	***	ns
*S × M × RB*				
MMS1 *×* 0 *×* RH	10.3 ± 1.0 f	32.1 ± 7.0	12.5 ± 2.2 k	51.0 ± 15.9
MMS1 *×* 0 *×* BK	10.4 ± 1.0 f	21.5 ± 8.0	12.6 ± 2.8 k	37.0 ± 12.6
MMS1 *×* 10 *×* RH	35.0 ± 2.2 e	96.4 ± 10.2	69.1 ± 4.0 h	146.4 ± 26.4
MMS1 *×* 10 *×* BK	43.1 ± 2.6 d	112.0 ± 15.3	59.6 ± 2.8 i	183.8 ± 43.9
MMS1 *×* 30 *×* RH	118.7 ± 5.1 b	181.2 ± 25.5	92.6 ± 3.4 f	483.6 ± 137.9
MMS1 *×* 30 *×* BK	122.7 ± 5.0 b	201.9 ± 35.5	78.0 ± 6.5 g	470.0 ± 138.8
MMS1 *×* 50 *×* RH	162.7 ± 5.0 a	287.0 ± 29.7	163.3 ± 8.6 b	843.0 ± 158.2
MMS1 *×* 50 *×* BK	120.2 ± 6.5 b	288.7 ± 32.8	134.0 ± 5.9 d	964.5 ± 128.3
LHS1 *×* 0 *×* RH	14.1 ± 4.2 g	44.0 ± 7.1	10.0 ± 4.7 k	63.4 ± 11.6
LHS1 *×* 0 *×* BK	11.5 ± 2.9 g	32.4 ± 7.1	10.4 ± 3.5 k	33.6 ± 11.7
LHS1 *×* 10 *×* RH	37.7 ± 2.0 e	119.7 ± 12.9	58.2 ± 1.5 i	140.2 ± 42.6
LHS1 *×* 10 *×* BK	43.5 ± 2.6 d	117.4 ± 11.7	42.9 ± 1.2 j	140.2 ± 48.5
LHS1 *×* 30 *×* RH	114.3 ± 5.2 b	216.0 ± 30.3	164.7 ± 5.6 b	618.8 ± 171.2
LHS1 *×* 30 *×* BK	94.7 ± 2.6 c	275.3 ± 48.0	106.1 ± 4.4 e	429.8 ± 121.9
LHS1 *×* 50 *×* RH	160.0 ± 4.9 a	355.0 ± 21.9	188.6 ± 5.8 a	1258.7 ± 202.1
LHS1 *×* 50 *×* BK	123.7 ± 5.8 b	321.8 ± 34.7	154.7 ± 4.4 c	1500.8 ± 165.0
	***	ns	***	ns
*S × M*	***	**	***	***

Non-significant (ns). **, *** significant at *p* ≤ 0.01, and 0.001, respectively. Simulants (S), amendment (M), and Rhizo vs. bulk soil (RB) and interaction were compared by Duncan’s multiple range test (*p* = 0.05). Different lowercase letters within each column indicate significant differences (*p* ≤ 0.05).

**Table 5:** Concentration (mg kg^−1^ DW) of main macro and micronutrients in different mixtures of MMS-1 or LHS-1 simulants and manure (simulant/manure rates: 100:0, 90:10, 70:30, 50:50; *w*/*w* %), separated in rhizo and bulk soil after lettuce growth, extracted by 1 M NH_4_NO_3_ (*n* = 3).

Source of Variance	Ca	K	Mg	P	Fe	Na	Mn	Cu	Zn
	mg kg^−1^ DW								
*Simulants (S)*									
MMS1	2215	768	416	11.8	0.69	45.2	1.57	0.15	0.10
LHS1	1102	490	246	8.46	1.45	69.2	0.67	0.17	0.13
	***	***	***	***	***	*	***	*	***
*Amendment % (M)*									
0	1201 d	90.3 d	156 d	0.10 d	0.04 c	31.3 b	0.24 b	0.04 d	0.03 d
10	1551 c	338 c	227 c	6.08 c	0.84 b	27.5 b	1.48 a	0.14 c	0.10 c
30	1869 b	707 b	388 b	14.9 b	1.52 a	39.4 b	1.39 a	0.19 b	0.14 b
50	2012 a	1382 a	553 a	19.5 a	1.87 a	130 a	1.37 a	0.27 a	0.19 a
	***	***	***	***	***	***	***	***	***
*Rhizo* vs. *bulk soil (RB)*									
RH	1634	527	303	9.82	1.12	51	1.13	0.16	0.11
BK	1683	731	359	10.5	1.01	63	1.11	0.16	0.12
	ns	**	***	ns	ns	ns	ns	ns	ns
*S × M × RB*									
MMS1 × 0 × RH	1950	147	260	0.13	0.04	27.3	0.08	0.06	0.02
MMS1 × 0 × BK	2124	187	296	0.13	0.04	36.9	0.06	0.06	0.03
MMS1 × 10 × RH	2258	421	334	7.39	0.19	29.5	2.22	0.11	0.06
MMS1 × 10 × BK	2176	558	375	9.28	0.59	30.8	2.39	0.12	0.09
MMS1 × 30 × RH	2339	671	406	18.1	0.85	29.0	2.01	0.17	0.11
MMS1 × 30 × BK	2183	1106	484	18.8	0.99	38.2	1.94	0.17	0.12
MMS1 × 50 × RH	2358	1254	528	19.5	1.61	66.3	2.00	0.25	0.16
MMS1 × 50 × BK	2330	1801	644	21.4	1.18	103	1.87	0.23	0.17
LHS1 × 0 × RH	330	15.9	45.8	0.09	0.04	29.7	0.45	0.03	0.03
LHS1 × 0 × BK	398	12.0	24.4	0.05	0.03	31.4	0.39	0.03	0.03
LHS1 × 10 × RH	796	147	78.4	2.80	1.16	26.7	0.58	0.13	0.08
LHS1 × 10 × BK	976	225	122	4.87	1.42	23.1	0.72	0.19	0.16
LHS1 × 30 × RH	1377	472	289	12.5	2.59	36.8	0.85	0.23	0.18
LHS1 × 30 × BK	1576	579	372	10.4	1.65	53.8	0.77	0.18	0.15
LHS1 × 50 × RH	1663	1092	486	18.1	2.52	163	0.88	0.30	0.22
LHS1 × 50 × BK	1697	1379	553	18.9	2.17	189	0.71	0.28	0.20
	ns	ns	ns	ns	ns	ns	ns	ns	ns
*S × M*	***	ns	***	***	*	**	***	**	ns

For the sake of clarity, this wide table shows only the mean values, not followed by standard deviations. Non-significant (ns). *, **, *** significant at *p* ≤ 0.05, 0.01, and 0.001, respectively. Simulants (S), amendment (M), and Rhizo vs. bulk soil (RB) and interaction were compared by Duncan’s multiple range test (*p* = 0.05). Different lowercase letters within each column indicate significant differences (*p* ≤ 0.05).

**Table 6:** Mineral contents in different mixtures of MMS-1 or LHS-1 simulants and manure (simulant/manure rates: 100:0, 90:10, 70:30, 50:50; *w*/*w* %).

Source of Variance	PO_4_	K	Mg	Ca	SO_4_	Na
	g kg^−1^ DW					
*Simulants (S)*						
MMS1	4.10 ± 0.73	26.53 ± 2.97	6.32 ± 0.60	1.69 ± 0.14	0.72 ± 0.14	1.89 ± 0.20
LHS1	3.54 ± 0.59	24.82 ± 1.79	5.69 ± 0.43	2.10 ± 0.11	0.66 ± 0.08	2.17 ± 0.37
	ns	ns	*	***	ns	ns
*Amendment % (M)*						
0	0.53 ± 0.02 c	16.01 ± 0.33 c	3.65 ± 0.15 c	1.65 ± 0.11 bc	0.21 ± 0.02 c	1.41 ± 0.21 b
10	3.89 ± 0.15 b	24.50 ± 0.93 b	5.78 ± 0.28 b	1.55 ± 0.10 c	0.67 ± 0.09 b	1.48 ± 0.19 b
30	4.67 ± 0.21 b	25.94 ± 0.34 b	7.04 ± 0.42 a	1.90 ± 0.19 b	0.72 ± 0.03 b	1.83 ± 0.27 b
50	6.19 ± 0.64 a	36.25 ± 3.33 a	7.56 ± 0.61 a	2.49 ± 0.11 a	1.14 ± 0.13 a	3.41 ± 0.33 a
	***	***	***	***	***	***
*S × M*						
MMS1 × 0	0.56 ± 0.02	15.93 ± 0.26 d	3.49 ± 0.21 e	1.44 ± 0.12	0.20 ± 0.02 d	1.21 ± 0.29 d
MMS1 × 10	3.83 ± 0.29	23.07 ± 1.19 b	6.29 ± 0.35 bcd	1.35 ± 0.02	0.50 ± 0.11 c	1.47 ± 0.24 cd
MMS1 × 30	5.07 ± 0.12	25.32 ± 0.43 bc	6.64 ± 0.67 bc	1.54 ± 0.13	0.78 ± 0.03 b	2.18 ± 0.00 bc
MMS1 × 50	6.95 ± 0.82	41.80 ± 3.59 a	8.87 ± 0.18 a	2.45 ± 0.13	1.40 ± 0.11 a	2.70 ± 0.16 b
LHS1 × 0	0.51 ± 0.04	16.10 ± 0.68 d	3.80 ± 0.21 e	1.85 ± 0.07	0.23 ± 0.03 d	1.61 ± 0.30 cd
LHS1 × 10	3.95 ± 0.15	25.93 ± 0.93 bc	5.27 ± 0.12 d	1.76 ± 0.07	0.85 ± 0.04 b	1.50 ± 0.35 cd
LHS1 × 30	4.26 ± 0.18	26.56 ± 0.06 bc	7.44 ± 0.53 b	2.26 ± 0.18	0.67 ± 0.04 bc	1.47 ± 0.48 cd
LHS1 × 50	5.43 ± 0.90	30.69 ± 3.39 b	6.25 ± 0.29 cd	2.53 ± 0.21	0.88 ± 0.10 b	4.11 ± 0.18 a
	ns	**	***	ns	***	*

Non-significant (ns). *, **, *** significant at *p* ≤ 0.05, 0.01, and 0.001, respectively. Simulants (S), amendment (M), and Rhizo vs. bulk soil (RB) and interaction were compared by Duncan’s multiple range test (*p* = 0.05). Different lowercase letters within each column indicate significant differences (*p* ≤ 0.05).

## Data Availability

The datasets generated for this study are available on request to the corresponding author.

## Supplementary Materials

The following supporting information can be downloaded at: https://www.mdpi.com/article/10.3390/plants11233345/s1, Figure S1: Interaction of simulant × manure concentration on percentage of total root length in each of ten diameter classes: a: D <= 0.5 mm; b: 0.5 < D<= 1 mm; c 1 < D<= 1.5 mm; d 1.5 < D<= 4.5 mm; e D > 4 mm. Orange bars: MMS1; blue bars: LHS1. Bars with different letters are different for *p* < 0.05 at the post hoc Duncan Multiple Range Test for *p* ≤ 0.05; Table S1: Summary of mineralogical and elemental composition and main physico-hydraulic and chemical properties of MMS-1 and LHS-1 simulants, horse/swine monogastric manure, and mixtures of MMS-1 or LHS-1 simulants and manure (simulant/manure rates: 100:0, 90:10, 70:30, 50:50; *w*/*w* %); Table S2: Concentration (mg kg^−1^ DW) of main macro and micronutrients in different mixtures of MMS-1 or LHS-1 simulants and manure (simulant/manure rates: 100:0, 90:10, 70:30, 50:50; *w*/*w* %), separated in rhizo and bulk soil after lettuce growth, extracted by 0.05 M EDTA at pH 7 (*n* = 3); Table S3: NH_4_NO_3_-extractable fraction (expressed as % of the total content) of main macro and micronutrients in different mixtures of MMS-1 or LHS-1 simulants and manure (simulant/manure rates: 100:0, 90:10, 70:30, 50:50; *w*/*w* %), separated in rhizo and bulk soil after lettuce growth (*n* = 3); Table S4: EDTA-extractable fraction (expressed as % of the total content) of main macro and micronutrients in different mixtures of MMS-1 or LHS-1 simulants and manure (simulant/manure rates: 100:0, 90:10, 70:30, 50:50; *w*/*w* %), separated in rhizo and bulk soil after lettuce growth (*n* = 3); Table S5: Nutrient uptake in different mixtures of MMS-1 or LHS-1 simulants and manure (simulant/manure rates: 100:0, 90:10, 70:30, 50:50; *w*/*w* %).
