# Supplementary material for: Can Lunar and Martian Soils Support Food Plant Production? Effects of Horse/Swine Monogastric Manure Fertilisation on Regolith Simulants Enzymatic Activity, Nutrient Bioavailability, and Lettuce Growth
Source: Plants (Basel). 2022 Dec 2;11(23):3345. doi: 10.3390/plants11233345 (PMC9740528; doi:10.3390/plants11233345)
Supplement: Supplementary file 1 [file plants-11-03345-s001.zip › plants-2023397-supplementary.pdf]

Can Lunar and Martian Soils Support Food Plant Production? Effects of Horse/Swine Monogastric Manure Fertilisation on Regolith Simulants Enzymatic Activity, Nutrient Bioavailability, and Lettuce Growth

Antonio G. Caporale [1,*,†], Mariana Amato [2,†], Luigi G. Duri [1], Rocco Bochicchio [2], Stefania De Pascale [1], Giuseppe Di Rauso Simeone [1], Mario Palladino [1], Antonio Pannico [1], Maria A. Rao [1], Youssef Rouphael [1,*] and Paola Adamo [1]

[1] Department of Agricultural Sciences, University of Naples Federico II, 80055 Portici, Italy
[2] School of Agriculture, Forestry, Food and Environmental Sciences, University of Basilicata, 85100 Potenza, Italy
* Correspondence: ag.caporale@unina.it (A.G.C.); youssef.rouphael@unina.it (Y.R.)
† These authors contributed equally to this work.

SUPPLEMENTARY MATERIAL

Table S1. Summary of mineralogical and elemental composition and main physico-hydraulic and chemical properties of MMS-1 and LHS-1 simulants, horse/swine monogastric manure, and mixtures of MMS-1 or LHS-1 simulants and manure (simulant/manure rates: 100:0, 90:10, 70:30, 50:50; w/w %)

	MMS-1	LHS-1	Horse/swine monogastric manure
Mineralogical composition	Plagioclase, amorphous minerals and zeolite (main), hematite and smectite (secondary)	Plagioclase and amorphous minerals (main), kaolinite and chlorite (secondary)	
Elemental composition	Si, Al, Fe, Ca, Mg, K, Na, P and Mn (macroelements), Ni, Cr, V, Zn, Cu and Pb (microelements)	Si, Al, Ca, Fe, Na, Mg, K, P, Mn and S (macroelements), Ni, Cr, V, Cu, Zn and Pb (microelements)	C, Ca, N, S, P, Fe, Mg, Na, Al, and Mn (macroelements), Zn, Cu, Cr, Pb, V and Ni (microelements)
Physico-hydraulic properties	Coarse textured substrate with high macroporosity and low water holding capacity	Coarse textured substrate with high macroporosity and low water holding capacity	Porous medium with high water holding capacity
Chemical properties	Alkaline pH (8.9), low EC and CEC, source of bioavailable Ca, Mg and K, low bioavailability of PTE	Alkaline pH (9.7), very low EC and CEC, source of bioavailable Ca, medium-low bioavailability of PTE	Alkaline pH (9.0), high EC and CEC, source of bioavailable K, Ca, Mg, N, P, Fe, S and Mn, medium-low bioavailability of PTE
MMS-1/manure mixtures (main properties)	Alkaline pH (8.9-9.0), low-to-high EC (0.3-3.3 dS m^{-1}), CEC (8-23 cmol+ kg^{-1}) and bioavailability of plant nutrients. Enhanced physico-hydraulic properties with increasing rates of manure in the mixture up to 30% in weight, then they got worst in the 50:50 w:w mixture. MMS-1/manure mixtures are better than LHS-1/manure ones in terms of chemical fertility (lower pHs and higher nutrient availability); this divergent fertility was particularly evident at 90:10 w:w rate and tended to be mitigated by increasing the levels of manure		
LHS-1/manure mixtures (main properties)	Alkaline pH (9.3-9.7), low-to-high EC (0.1-3.7 dS m^{-1}), CEC (1-22 cmol+ kg^{-1}) and bioavailability of plant nutrients. Enhanced physico-hydraulic properties with increasing rates of manure in the mixture up to 30% in weight, then they got worst in the 50:50 w:w mixture. LHS-1/manure mixtures are better than MMS-1/manure ones in terms of water retention, especially in the 'dry' region of matrix potential head (between -100 and -600 cm)		

The mineralogical and elemental compositions of two simulants were assessed by X-ray powder diffraction (XRPD), wavelength- and energy-dispersive X-ray fluorescence (WD- and ED-XRF), respectively. The elemental profile of horse/swine monogastric manure was determined by CHNS elemental analyser and inductively coupled plasma - optical emission spectrometry (ICP-OES), after acid digestion in a microwave-assisted digestion system. Physico-hydraulic and chemical properties were assessed according to internationally harmonised and standardised analytical protocols for characterisation of soil/substrate samples. EC indicates electrical conductivity, CEC: cation exchange capacity and PTE: potentially toxic elements.

Table S2. Concentration (mg kg^{-1} DW) of main macro and micronutrients in different mixtures of MMS-1 or LHS-1 simulants and manure (simulant/manure rates: 100:0, 90:10, 70:30, 50:50; w/w %), separated in rhizo and bulk soil after lettuce growth, extracted by 0.05M EDTA at pH 7 (n=3).

Source of Variance	Ca	K	Mg	P	Fe	Na	Mn	Cu	Zn
	mg kg^{-1} DW								
Simulants (S)									
MMS1	11756	770	772	687	222	119	115	6.03	21.5
LHS1	7981	460	514	556	356	150	45.5	6.00	21.1
	***	***	***	***	***	**	***	ns	ns
Amendment % (M)									
0	2794 d	85.3 d	235 d	124 d	18.0 c	87.8 c	12.9 d	0.72 d	1.13 d
10	6659 c	333 c	418 c	404 c	230 b	93.8 c	81.0 c	4.75 c	10.0 c
30	13981 b	715 b	863 b	934 b	452 a	131 b	109 b	8.42 b	29.0 b
50	16039 a	1326 a	1055 a	1025 a	455 a	226 a	118 a	10.2 a	45.1 a
	***	***	***	***	***	***	***	***	***
Rhizo vs bulk soil (RB)									
RH	9933	521	598	626	297	129	80.0	6.30	21.6
BK	9803	709	687	617	280	140	80.7	5.73	21.1
	ns	***	***	ns	ns	ns	ns	**	ns
S x M x RB									
MMS1 x 0 x RH	5065	159	420	245	14.8	83.2	25.8 f	0.80	2.14
MMS1 x 0 x BK	5244	169	445	240	9.77	88.4	21.2 f	0.70	1.09
MMS1 x 10 x RH	8753	389	555	477	107	93.2	131 c	4.02	9.28
MMS1 x 10 x BK	9135	548	662	491	115	96.9	146 b	4.02	9.72
MMS1 x 30 x RH	15781	678	884	980	353	111	151 ab	9.15	29.0
MMS1 x 30 x BK	15759	1133	1050	994	361	123	158 a	8.55	29.6
MMS1 x 50 x RH	17170	1270	950	1062	417	157	146 b	11.50	47.3
MMS1 x 50 x BK	17139	1815	1206	1006	395	202	143 b	9.48	44.0
LHS1 x 0 x RH	430	8.65	47.4	5.74	23.2	89.9	2.28 g	0.67	0.66
LHS1 x 0 x BK	435	4.29	25.7	4.15	24.1	89.5	2.37 g	0.69	0.64
LHS1 x10 x RH	4607	173	209.2	338	367	93.2	24.4 f	5.38	11.16
LHS1 x 10 x BK	4142	223	246	311	332	91.8	22.9 f	5.58	9.95
LHS1 x 30 x RH	12267	465	703	899	567	136	66.2 e	8.48	28.7
LHS1 x 30 x BK	12117	586	815	862	526	153	62.1 e	7.50	28.8
LHS1 x 50 x RH	15394	1023	1017	1003	526	268	93.1 d	10.4	44.1
LHS1 x 50 x BK	14452	1196	1047	1029	480	276	90.5 d	9.31	45.0
	ns	ns	ns	ns	ns	ns	*	ns	ns
S x M	***	ns	***	***	***	*	***	***	ns

*For the sake of clarity, this wide table shows only the mean values, not followed by standard deviations. Non-significant (ns). *, **, *** Significant at P ≤ 0.05, 0.01, and 0.001, respectively. Simulants (S), Amendment (M) and Rhizo vs bulk soil (RB) and interaction were compared by Duncan's multiple-range test (P = 0.05). Different lowercase letters within each column indicate significant differences (P ≤ 0.05).*

Table S3. NH_4NO_3-extractable fraction (expressed as % of the total content) of main macro and micronutrients in different mixtures of MMS-1 or LHS-1 simulants and manure (simulant/manure rates: 100:0, 90:10, 70:30, 50:50; w/w %), separated in rhizo and bulk soil after lettuce growth (n=3).

Source of Variance	Ca	K	Mg	P	Fe	Na	Mn	Cu	Zn
	% of the total content								
Simulants (S)									
MMS1	5.2	4.4	2.2	0.41	0.002	0.20	0.22	0.32	0.11
LHS1	2.4	2.8	1.4	0.27	0.003	0.34	0.09	0.37	0.16
	***	***	***	***	**	***	***	*	**
Amendment (M)									
0	3.4 c	0.5 d	0.6 d	0.01 d	<0.001 c	0.10 c	0.03 b	0.14 c	0.06 b
10	4.0 a	1.9 c	1.0 c	0.39 c	0.001 c	0.10 c	0.20 a	0.38 b	0.15 a
30	4.0 a	4.1 b	2.0 b	0.51 a	0.003 b	0.17 b	0.20 a	0.40 b	0.16 a
50	3.7 b	7.9 a	3.6 a	0.45 b	0.005 a	0.72 a	0.21 a	0.46 a	0.17 a
	*	***	***	***	*	***	**	***	**
Rhizo vs bulk soil (RB)									
RH	3.7	3.0	1.6	0.32	0.003	0.24	0.16	0.34	0.12
BK	3.9	4.2	2.0	0.36	0.002	0.30	0.16	0.35	0.14
	ns	***	**	**	*	***	ns	ns	*
S x M x RB									
MMS1 x 0 x RH	5.6	0.8 h	1.1	0.01	<0.001 e	0.09	0.01	0.19 d	0.05 f
MMS1 x 0 x BK	6.1	1.1 h	1.2	0.01	<0.001 e	0.12	0.01	0.19 d	0.06 f
MMS1 x 10 x RH	5.8	2.4 g	1.5	0.47	<0.001 e	0.10	0.30	0.31 c	0.09 e
MMS1 x 10 x BK	5.6	3.2 ef	1.6	0.59	0.001 de	0.11	0.32	0.34 c	0.14 cd
MMS1 x 30 x RH	5.1	3.8 e	2.1	0.61	0.002 d	0.12	0.29	0.37 c	0.12 d
MMS1 x 30 x BK	4.7	6.3 d	2.5	0.64	0.002 d	0.16	0.28	0.36 c	0.14 cd
MMS1 x 50 x RH	4.4	7.2 c	3.4	0.45	0.005 b	0.36	0.31	0.43 b	0.14 cd
MMS1 x 50 x BK	4.3	10.3 a	4.1	0.49	0.003 c	0.57	0.29	0.39 bc	0.16 c
LHS1 x 0 x RH	0.9	0.1 i	0.2	0.01	<0.001 e	0.10	0.06	0.09 e	0.06 f
LHS1 x 0 x BK	1.1	0.1 i	0.1	0.01	<0.001 e	0.10	0.05	0.10 e	0.06 f
LHS1 x10 x RH	2.1	0.8 h	0.3	0.18	0.002 c	0.09	0.08	0.35 c	0.12 d
LHS1 x 10 x BK	2.5	1.3 h	0.5	0.31	0.002 c	0.08	0.10	0.51 a	0.25 a
LHS1 x 30 x RH	3.0	2.7 fg	1.5	0.42	0.006 ab	0.16	0.12	0.48 a	0.20 b
LHS1 x 30 x BK	3.4	3.3 ef	1.9	0.35	0.004 bc	0.23	0.11	0.38 bc	0.17 c
LHS1 x 50 x RH	3.1	6.3 d	3.1	0.42	0.007 a	0.90	0.14	0.53 a	0.20 b
LHS1 x 50 x BK	3.2	7.9 b	3.6	0.44	0.006 ab	1.04	0.11	0.49 a	0.18 bc
	ns	*	ns	ns	**	ns	ns	**	**
S x M	***	**	***	***	**	***	**	***	***

*For the sake of clarity, this wide table shows only the mean values, not followed by standard deviations. Non-significant (ns). *, **, *** Significant at P ≤ 0.05, 0.01, and 0.001, respectively. Simulants (S), Amendment (M) and Rhizo vs bulk soil (RB) and interaction were compared by Duncan's multiple-range test (P = 0.05). Different lowercase letters within each column indicate significant differences (P ≤ 0.05).*

Table S4. EDTA-extractable fraction (expressed as % of the total content) of main macro and micro-nutrients in different mixtures of MMS-1 or LHS-1 simulants and manure (simulant/manure rates: 100:0, 90:10, 70:30, 50:50; w/w %), separated in rhizo and bulk soil after lettuce growth (n=3).

Source of Variance	Ca	K	Mg	P	Fe	Na	Mn	Cu	Zn
	% of the total content								
Simulants (S)									
MMS1	25.9	4.4	4.1	29.0	0.5	0.5	16.5	12.6	23.0
LHS1	16.7	2.6	2.9	18.7	0.8	0.7	6.7	12.8	22.6
	***	***	***	***	***	**	***	ns	ns
Amendment (M)									
0	8.0 c	0.5 d	0.9 d	14.2 d	<0.1 d	0.3 c	1.7 d	2.3 c	2.2 d
10	17.2 b	1.9 c	1.8 c	25.9 b	0.4 c	0.3 c	10.8 c	13.1 b	15.7 c
30	30.2 a	4.1 b	4.5 b	31.7 a	1.0 b	0.6 b	15.7 b	17.9 a	33.0 b
50	29.8 a	7.6 a	6.8 a	23.7 c	1.3 a	1.2 a	18.2 a	17.6 a	40.3 a
	***	***	***	***	***	***	***	***	***
Rhizo vs bulk soil (RB)									
RH	21.4	3.0	3.3	24.0	0.7	0.6	11.5	13.2	23.1
BK	21.2	4.1	3.8	23.7	0.7	0.6	11.6	12.2	22.5
	ns	***	**	ns	ns	ns	ns	**	ns
S x M x RB									
MMS1 x 0 x RH	14.5	0.9 i	1.7	28.1	<0.1	0.3	3.3	2.6	4.1
MMS1 x 0 x BK	15.0	1.0 i	1.8	27.5	<0.1	0.3	2.7	2.3	2.1
MMS1 x 10 x RH	22.6	2.2 h	2.4	30.5	0.2	0.3	17.5	11.1	14.5
MMS1 x 10 x BK	23.6	3.1 fg	2.9	31.4	0.2	0.3	19.5	11.1	15.2
MMS1 x 30 x RH	34.1	3.9 e	4.6	33.3	0.8	0.5	21.6	19.4	33.0
MMS1 x 30 x BK	34.1	6.5 c	5.5	33.7	0.8	0.5	22.6	18.2	33.7
MMS1 x 50 x RH	31.9	7.3 b	6.1	24.6	1.2	0.9	22.5	19.9	42.3
MMS1 x 50 x BK	31.9	10.4 a	7.7	23.3	1.1	1.1	22.0	16.4	39.3
LHS1 x 0 x RH	1.2	<0.1 j	0.2	0.7	<0.1	0.3	0.3	2.2	1.3
LHS1 x 0 x BK	1.2	<0.1 j	0.1	0.5	<0.1	0.3	0.3	2.2	1.2
LHS1 x10 x RH	11.9	1.0 i	0.9	21.6	0.6	0.3	3.3	14.8	17.5
LHS1 x 10 x BK	10.7	1.3 i	1.1	19.9	0.6	0.3	3.1	15.3	15.5
LHS1 x 30 x RH	26.5	2.7 gh	3.7	30.5	1.2	0.6	9.5	18.0	32.7
LHS1 x 30 x BK	26.2	3.4 ef	4.2	29.3	1.1	0.7	8.9	15.9	32.8
LHS1 x 50 x RH	28.6	5.9 d	6.5	23.2	1.5	1.5	14.4	17.9	39.5
LHS1 x 50 x BK	26.9	6.8 bc	6.7	23.8	1.4	1.5	14.0	16.1	40.3
	ns	**	ns	ns	ns	ns	ns	ns	ns
S x M	**	***	***	***	***	**	***	**	ns

*For the sake of clarity, this wide table shows only the mean values, not followed by standard deviations. Non-significant (ns). *, **, *** Significant at P ≤ 0.05, 0.01, and 0.001, respectively. Simulants (S), Amendment (M) and Rhizo vs bulk soil (RB) and interaction were compared by Duncan's multiple-range test (P = 0.05). Different lowercase letters within each column indicate significant differences (P ≤ 0.05).*

Table S5. Nutrient uptake in different mixtures of MMS-1 or LHS-1 simulants and manure (simulant/manure rates: 100:0, 90:10, 70:30, 50:50; w/w %).

Source of Variance	PO_4	K	Mg	Ca	SO_4	Na
	mg plant^{-1}					
Simulants (S)						
MMS1	21.2 ± 3.97	121 ± 20.3	7.20 ± 1.160	29.7 ± 5.04	3.48 ± 0.69	8.72 ± 1.61
LHS1	5.54 ± 1.41	35.9 ± 8.7	2.76 ± 0.612	8.40 ± 2.04	1.02 ± 0.27	2.34 ± 0.53
	***	***	***	***	***	***
Amendment % (M)						
0	0.24 ± 0.04 c	6.92 ± 1.1 c	0.68 ± 0.071 d	1.55 ± 0.23 d	0.09 ± 0.01 b	0.56 ± 0.08 c
10	16.9 ± 3.32 b	104 ± 18.3 a	6.38 ± 0.883 b	25.9 ± 5.56 b	2.65 ± 0.44 a	6.55 ± 1.53 b
30	22.2 ± 5.73 a	117 ± 26.2 a	7.74 ± 1.250 a	30.9 ± 6.55 a	3.41 ± 0.88 a	9.10 ± 2.68 a
50	14.1 ± 5.52 b	84.9 ± 33.2 b	5.14 ± 1.883 c	18.0 ± 6.99 c	2.84 ± 1.16 a	5.89 ± 1.89 b
	***	***	***	***	***	***
S x M						
MMS1 x 0	0.32 ± 0.04 d	9.19 ± 1.1 d	0.82 ± 0.053 d	2.01 ± 0.24 e	0.11 ± 0.01 e	0.66 ± 0.10 d
MMS1 x 10	23.6 ± 2.93 b	141 ± 14.1 b	8.22 ± 0.492 b	38.2 ± 0.81 b	3.12 ± 0.82 b	9.11 ± 1.94 b
MMS1 x 30	34.9 ± 1.54 a	174 ± 9.8 a	10.5 ± 0.350 a	45.3 ± 2.25 a	5.35 ± 0.39 a	15.0 ± 0.68 a
MMS1 x 50	26.1 ± 2.94 b	157 ± 15.4 ab	9.26 ± 0.871 b	33.4 ± 2.35 c	5.33 ± 0.70 a	10.1 ± 0.14 b
LHS1 x 0	0.15 ± 0.02 d	4.66 ± 0.4 d	0.54 ± 0.046 d	1.10 ± 0.10 e	0.07 ± 0.01 e	0.47 ± 0.10 d
LHS1 x 10	10.3 ± 1.57 c	67.4 ± 10.0 c	4.53 ± 0.500 c	13.6 ± 1.34 d	2.19 ± 0.28 bc	3.99 ± 1.21 c
LHS1 x 30	9.57 ± 1.29 c	59.2 ± 5.4 c	4.97 ± 0.047 c	16.4 ± 0.73 d	1.47 ± 0.04 cd	3.23 ± 0.93 cd
LHS1 x 50	2.15 ± 0.23 d	12.2 ± 0.6 d	1.02 ± 0.095 d	2.52 ± 0.16 e	0.35 ± 0.02 de	1.66 ± 0.10 cd
	***	***	***	***	***	***

Non-significant (ns). *, **, *** Significant at $P \leq 0.05$, 0.01, and 0.001, respectively. Simulants (S), Amendment (M) and Rhizo vs bulk soil (RB) and interaction were compared by Duncan's multiple-range test ($P = 0.05$). Different lowercase letters within each column indicate significant differences ($P \leq 0.05$).

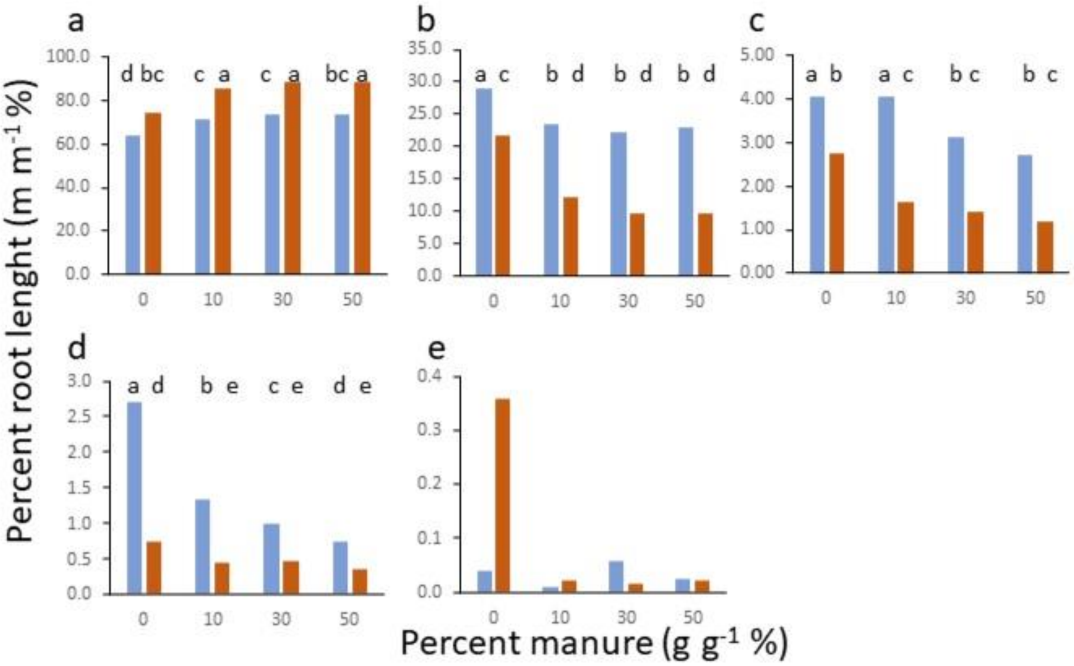

Figure S1. Interaction of simulant x manure concentration on percentage of total root length in each of ten diameter classes: a: D<= 0.5 mm; b: 0.5<D<=1 mm; c 1<D<=1.5 mm; d 1.5<D<=4.5 mm; e D> 4 mm. Orange bars: MMS1; blue bars: LHS1. Bars with different letters are different for P<0.05 at the post-hoc Duncan Multiple Range Test for p ≤ 0.05.
